# Unravelling the structure of CO_2_ in silica adsorbents: an NMR and computational perspective

**DOI:** 10.1039/d3cc05942a

**Published:** 2024-03-15

**Authors:** Mariana Sardo, Tiago Morais, Márcio Soares, Ricardo Vieira, Marina Ilkaeva, Mirtha A. O. Lourenço, Ildefonso Marín-Montesinos, Luís Mafra

**Affiliations:** a CICECO – Aveiro Institute of Materials, Department of Chemistry, University of Aveiro 3810-193 Aveiro Portugal lmafra@ua.pt; b Department of Chemical and Environmental Engineering, University of Oviedo Av. Julián Clavería 8 33006 Oviedo Spain; c Department of Chemistry, University of Iceland, Science Institute Dunhaga 3 107 Reykjavik Iceland

## Abstract

This comprehensive review describes recent advancements in the use of solid-state NMR-assisted methods and computational modeling strategies to unravel gas adsorption mechanisms and CO_2_ speciation in porous CO_2_-adsorbent silica materials at the atomic scale. This work provides new perspectives for the innovative modifications of these materials rendering them more amenable to the use of advanced NMR methods.

## Abstract 2

Recent advancements in the combined use of solid-state NMR-assisted methods and computational modeling strategies are highlighted to unravel gas adsorption mechanisms, and CO_2_ speciation in porous CO_2_-adsorbent silica materials at the atomic scale.

## Graphical abstract

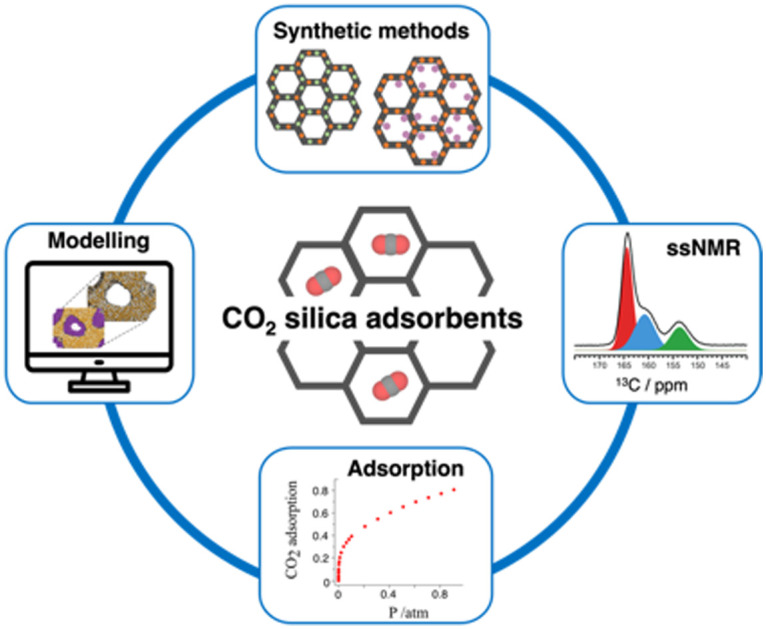

## 1. Introduction

### 1.1. Environmental challenges and the development of new sorbent materials for carbon capture

Global warming will continue to increase in the next decades (2021–2040) mainly due to increased cumulative CO_2_ emissions in nearly all considered scenarios.^1,2^ 80% of the global energy supply still relies on the consumption of fossil fuels, responsible for the increase by almost 2.1 Gt of energy-related CO_2_ emissions in 2021, making it the largest ever year-on-year increase in energy-related CO_2_ emissions in absolute terms.^3^ From 2010–2019, the average annual global greenhouse gas emissions were at their highest level in human history, but the rate of growth has slowed down. Without an immediate reduction in emission levels, across all sectors, limiting global warming to 1.5 °C will not be achieved in the early 2030s.^4^ To address this, it is crucial to achieve net-zero emissions. The Intergovernmental Panel for Climate Change (IPCC) suggests that agriculture, forestry, and other land use may provide large-scale emission reduction by removing and storing CO_2_. However, land cannot compensate for delayed emissions reductions in other sectors.^1,2^ CO_2_ capture is thus an imperative, which has led to the exploration of various carbon capture technologies. The concentration of CO_2_ in the gas stream, the pressure of the gas stream and the fuel type (solid or gaseous) are important factors in selecting the capture system.

There are three main approaches to CO_2_ capture, for industrial and power plant applications. Post-combustion^5^ systems separate CO_2_ from the flue gases produced by combustion of a primary fuel (coal, natural gas, oil or biomass) in air. Oxy-fuel combustion^6^ uses oxygen instead of air for combustion, producing a flue gas that is mainly composed of H_2_O and CO_2_ and therefore readily captured. Pre-combustion^7^ systems process the primary fuel in a reactor to produce separate streams of CO_2_ for storage and H_2_ which is used as a fuel. Other industrial processes, including the production of low-carbon or carbon-free fuels, employ one or more of these basic capture methods.^2^ Post-combustion capture is the most popular and mature technology used in the industry, as it requires no modification of existing plants. It includes absorption, adsorption, cryogenic, membrane and hydrate-based separations.^8^

Adsorption is especially significant as it is capable of separating CO_2_ from various mixtures such as flue gas (CO_2_/N_2_, CO_2_/H_2_) and biogas (CO_2_/CH_4_). Previous studies have shown that amine-based systems for CO_2_ absorption are the most suitable for combustion-based power plants for several reasons. These systems are highly efficient for diluted CO_2_ streams (*e.g.*, flue gases from coal combustion with *ca.* 10% to 12% CO_2_ by volume) are currently available and used at the commercial level and bear similarities to other “end-of-pipe” environmental control systems employed in power plants, operating under normal temperature and pressure conditions.^9^

Aqueous solutions of monoethanolamine have been the benchmark technology for scrubbing CO_2_ from process gases,^10,11^ due to their intrinsic high affinity towards CO_2_ and remain the standard to which other technologies are compared. However, they present high energy consumption requirements and operational issues such as degradation and toxicity. To overcome such limitations, several classes of solid amine adsorbents have emerged, as they combine the advantages of amine solutions and solid porous materials. Due to the high reactivity between loaded amines and CO_2_, solid amine adsorbents can selectively adsorb CO_2_ efficiently and quickly, while the CO_2_ regeneration energy decreases by more than 50% compared to amine solutions.^12^ These solid adsorbents include activated carbons, ion-exchange resins, zeolites, porous silicates, metal oxides, organic–inorganic hybrid sorbents (*e.g.*, supported amine-modified silicas, metal–organic frameworks (MOFs)), covalent–organic frameworks (COFs), and composite materials.^13–27^

### 1.2. Silica materials for CO_2_ capture applications

Silica sorbents are highly promising in the pursuit of mitigating climate change, particularly in carbon capture for industrial applications, as reported in the extensive reviews dedicated to the topic.^8,28^

Mesoporous silica is a silicon and oxygen-based porous material with pores ranging from 2–50 nm in diameter, making it ideal for the capture and storage of CO_2_. Its high surface area and large pore volume enable efficient CO_2_ adsorption, while its chemical stability and resistance to degradation (due to the absence of organic moieties in the structure) make it suitable for use under harsh industrial conditions (*e.g.* high pressure and temperature). The tuneable pore size and ordered mesoporous structure greatly enhance adsorption performance, while abundant surface silanol groups facilitate the framework modification with amine moieties.^29^ Amine modified silica-based materials show promising capture capacity at moderate temperatures (40–80 °C), and a relatively low desorption temperature, which decreases the energy cost of regeneration.^30^ Hence, silica-based materials are widely used and have been the subject of extensive research. To capture CO_2_ using mesoporous silica, the gas is typically passed through a fixed-bed reactor with the material, where it is adsorbed onto the surface of the pores and then heated to release the CO_2_ for storage or further use. However, the cost of the material and adsorption capacity is currently a bottleneck in using mesoporous silica for CO_2_ capture. Additionally, different preparation methods can influence amine loading, thermal stability, and adsorption capacity, which should be selected based on the application. Many researchers are seeking ways to lower costs and improve efficiency, such as using cheaper precursors or developing new, more efficient adsorption materials.

### 1.3. Which analytical tools are more promising to characterise CO_2_ adsorbent materials?

Characterising complex adsorbent materials for carbon capture is challenging and often limits the design of improved materials. Currently, various techniques such as single-crystal and powder X-ray diffraction, infrared and NMR spectroscopies are used to understand CO_2_ capture, each with its own advantages and limitations.^31^ Solid-state (ss) NMR spectroscopy is an attractive and site-selective technique for investigating CO_2_ binding modes in adsorbents as it provides atomic-scale insights into the type of chemical species formed upon CO_2_ adsorption, the local structure, dynamics and diffusion of CO_2_ species formed at the surface of the sorbent material, without the need for long-range ordering (necessary for X-ray diffraction methods), shedding light on the type of gas-sorption surface mechanisms.

This review aims to summarize the most relevant contributions published in the field of silica-based CO_2_ sorbent materials wherein ssNMR has been explored to investigate at the atomic level the structure and molecular dynamics of CO_2_ species formed at porous surfaces. Following this general introduction, in which we have provided the motivation underlying the current focus on studying CO_2_ sorbents based on silica materials, from an ssNMR perspective, the remainder of this feature article is organized into four core sections (2–5) wherein we try to maintain the focus on our research while always trying to make bridges with related works from other authors whenever pertinent. Section 2 describes the available methods applied in the chemical modification of silica materials, including a comparative overview of various modified silica materials with respect to the type of amine, amine density, textural properties, and CO_2_ capture capabilities. A perspective view of the strategies for the surface modification of silica materials for enhancing NMR signals is also included. Section 3 focuses on the study of CO_2_ adsorption mechanisms through the combination of ssNMR methods and computer modelling. CO_2_ adsorption studies performed by ssNMR-assisted techniques are reported in Section 4. Section 5 is dedicated to modelling and simulation approaches applied to micro and mesostructured materials and although we have only provided residual contributions here, we nevertheless attempted to gather topics that are still underexplored or not yet widely used in these materials, including *in silico* functionalization strategies and computational methods to model pore surface interactions with adsorbates. Section 6 provides a brief overview of the potential applications of CO_2_ silica chemisorbents and what role may ssNMR have in the design of better and more efficient sorbents.

## 2. Silica surface modification strategies for CO_2_ capture applications

### 2.1. Ordered mesoporous silica-based materials

In 1990, the first periodic mesoporous silica (PMS) materials were discovered, the M41S family.^32,33^ Despite their interest, these materials had thin walls (0.3–1 nm) and small pores (2–10 nm),^34^ resulting in poor hydrothermal and mechanical stability. In 1998, the Santa Barbara Amorphous (SBA) family materials^35,36^ addressed these limitations by exhibiting thicker pore walls (3–6 nm), larger pore sizes (5–30 nm) and a highly ordered mesoscale structure. This led to enhanced diffusion rates of molecules within the pores,^35,36^ making these materials appealing for incorporating organic functionalities and creating hybrid materials with distinct properties.

**Fig. 1:**
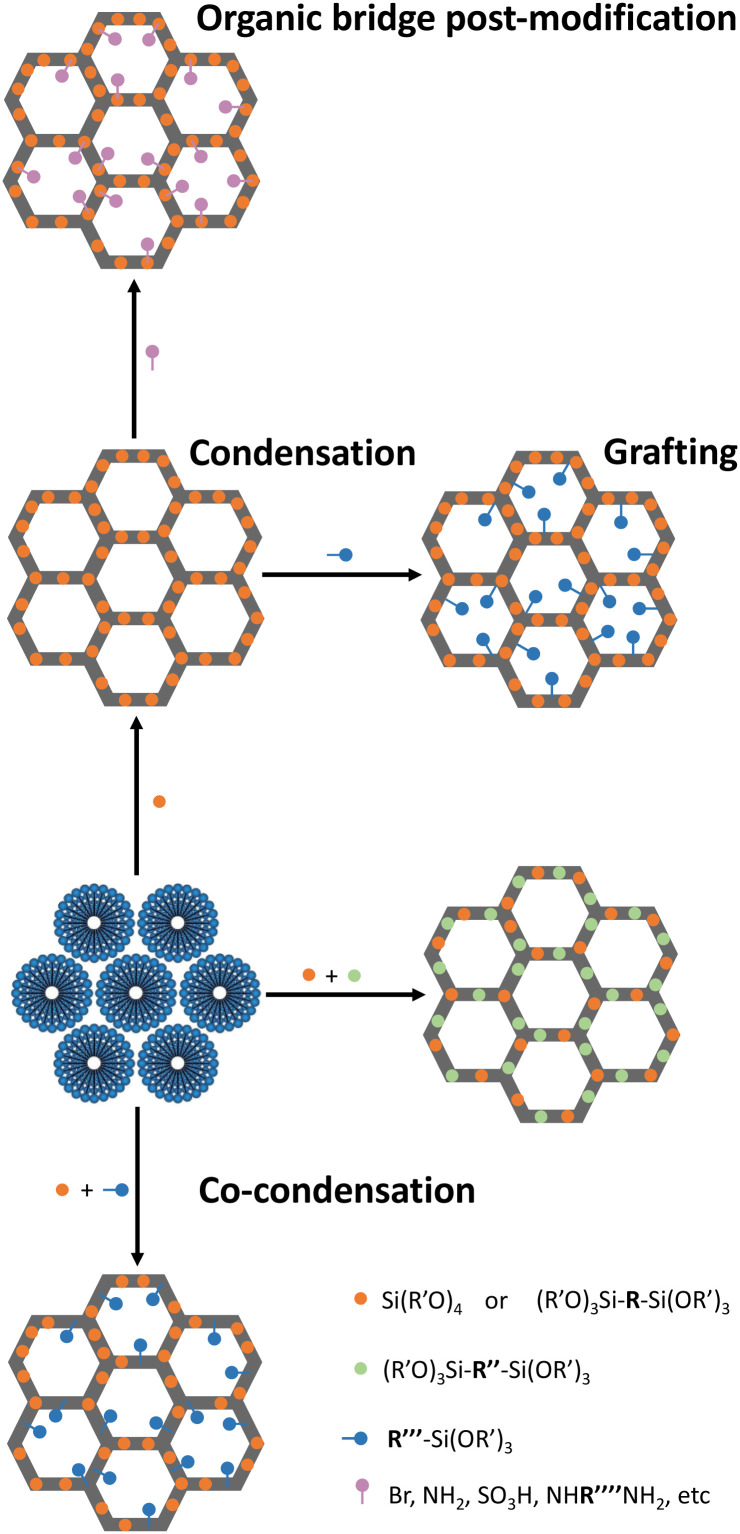
Schematic representation for the functionalization of PMS or PMO.

In 1999, a novel class of hybrid materials, known as periodic mesoporous organosilicas (PMO), was successfully synthesized.^37–39^ This family of silica-based materials allows the incorporation of high concentrations of organic linkers directly into their pore walls. Notably, a plethora of amine choices can be seamlessly integrated as an integral part of the inorganic-oxide framework. This integration can enhance the formation of CO_2_ adducts without compromising the textural properties of the adsorbent (*e.g.*, pore volume and surface area). This versatility of introducing functional groups (**R**) during the condensation reaction of organosilane precursors ((R′O)_3_Si-**R**-Si(OR′)_3_) effectively addresses challenges related to pore-blockage or excessive amine dilution commonly observed in the PMS sorbents. In PMO sorbents, by changing the size of the **R** group of the organosilane precursor, it is possible to control the functional group type and distance, the silanol density, the hydrophilicity/hydrophobicity, the rigidity/flexibility, and the mechanical/thermal/hydrothermal stability.^37–39^ Independently of the silica family, introducing organic moieties covalently bonded along the channel pore wall can be achieved through two main distinct methods: (i) co-condensation and (ii) post-modification reactions, which include grafting or anchoring strategies typically at the free silanol groups or at the organic bridge, in the case of PMOs (Fig. 1).^40^ The details about each method and limitations/advantages thereof are abundantly described elsewhere.^41,42^

These methods enable PMS or PMO materials’ tunability to achieve different polarities and/or introduce new reaction centres. These approaches have been employed to study the impact of factors such as amine loading, amine type, and inter-amine functional group distance in developing amine-modified porous silica (AMPS) sorbents specifically designed for CO_2_ adsorption–separation.^37–39^

### 2.2. Surface amine-modification towards enhanced CO_2_ capture

**Fig. 2:**
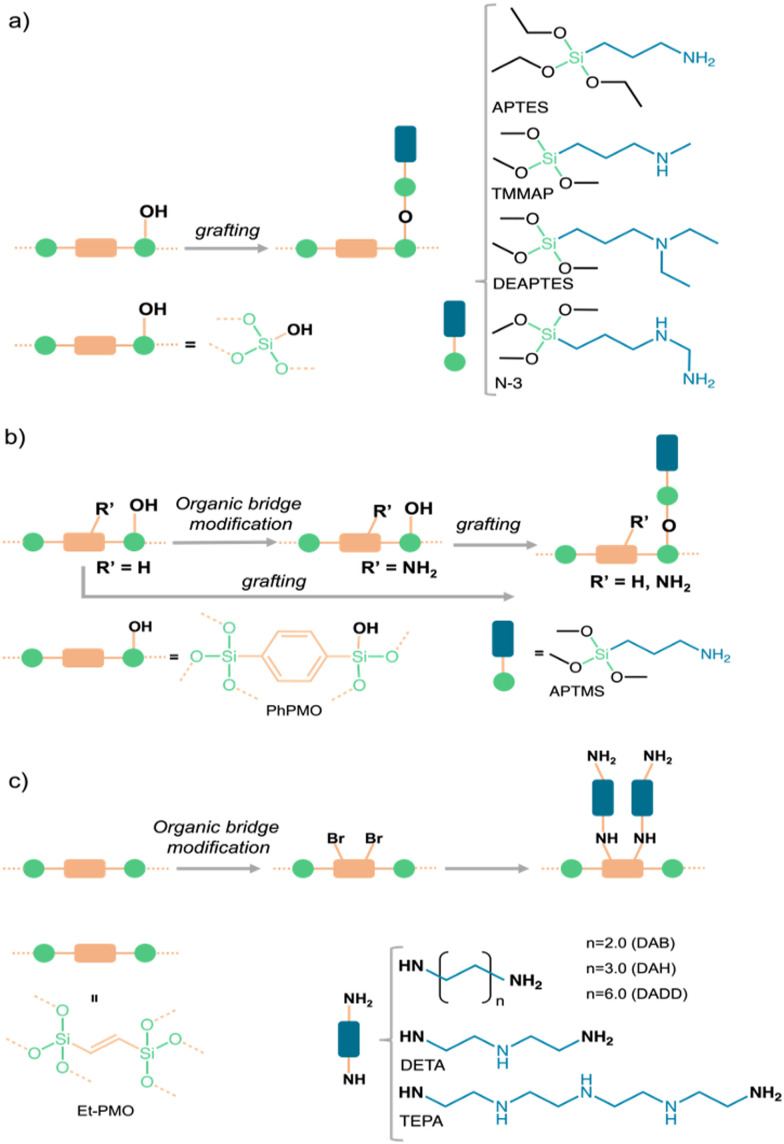
Schematic illustration of PMS or PMO modifications through (a, b) grafting and (b, c) organic-bridge functionalization, employing various amine sources.

Despite post-modification through grafting often results in a decrease in the specific surface area depending on the content and size of the group introduced (*cf.*, Table 1),^43^ this modification methodology is the most applied, because as opposed to the co-condensation method, the fact that the functionalization (grafting) occurs after PMS synthesis, it has minimal impact on mesostructure periodicity, often causing broad pore-size distributions, constraining the shape, size, and selectivity of these materials, as observed in CO_2_ sorbents prepared by co-condensation of bis((triethoxysilyl)propyl)ethylenediamine (BTEPED) and TEOS (Fig. 2 and Table 1).^44^

**Table 1:** CO_2_ adsorption capacities at 1 bar and textural properties for selected amine-modified based silicas

Sample type	Amine group/amine type	Amine loading (mmol g^−1^)	*S*_BET_ a (m^2^ g^−1^)	*V*_p_ b (cm^3^ g^−1^)	*d*_p_ c (nm)	CO_2_ ads. capacity (mmol g^−1^)	Ads. temp. (°C)	Ratio^d^ CO_2_ ads./N loading	Ratio CO_2_ ads/*S*_BET_ (mmol m^−2^ × 10^3^)
Co-condensation									
GD7^44^	BTEPED + TEOS/Sec.	n.p.	735	0.75	5.0	1.5	0	—	2.04
GD5^44^			727	0.58	2.0/5.0	1.9			2.61
GD3^44^			13	0.04	Disordered	2.1			161.54
GD0^44^	BTEPED/Sec.		38	0.10		2.3			60.52

Grafting									
MCM-41^45^	—	—	1088	0.83	2.6	0.37	45	—	0.34
PE-MCM-4^45^			894	1.28	5.1	0.34			0.38
PE-MCM-AP^45^	APTMS/Prim.	2.43	544	0.74	4.3	0.87		0.36	1.60
PE-MCM-ED^45^	*N*-3/Prim. + Sec.	4.00	421	0.60	4.2	1.51		0.38	3.59
PE-MCM-DT^45^	DT/Tert. Prim. + Sec.	4.86	373	0.54	4.0	1.75		0.36	4.69
SBA-15^46^	—	—	717	0.92	7.0	∼0.65	25	—	0.91
APTES@SBA-15^46^	APTES/Prim.	2.82	344	0.56	6.4	∼1.05		0.37	3.05
3-DEAPTES@SBA-15^46^	DEAPTES Tert.	1.59	318	0.46	5.8	∼0.25		0.16	0.79
TMMAP@SBA-15^46^	TMMAP/Sec.	3.09	260	0.39	5.9	∼0.85		0.28	3.27
*N*-3@SBA-15^46^	*N*-3/Prim. + Sec.	3.93	241	0.39	5.5	∼1.45		0.37	6.02
APTES@PhPMO^47^	APTES/Prim.	—	—	—	—	0.72		—	—
TSPED@Ph-PMO^47^	*N*-3/Prim. + Sec.	4.90	180	0.37	7.2	2.25		0.46	12.50
TEIPS@Ph-PMO^47^	TEIPS/Tert.	—	—	—	—	0.40		—	—
APTMS@PhPMO^48^	APTMS/Prim.	1.39	634	0.43	2.2	0.72		0.52	1.14

Organic bridge post-synthetic modification									
DAB-Et-PMO^49^	DAB/Prim. + Sec.	1.81	368	0.42	5.4	0.16^e^	0	0.09	60.53
DAH-Et-PMO^49^	DAH/Prim. + Sec.	0.60	449	0.56	5.2	0.13^e^		0.22	0.43
DADD-Et-PMO^49^	DADD/Prim. + Sec.	0.33	450	0.46	5.7	0.14^e^		0.42	0.29
DETA-Et-PMO^49^	DETA/Prim. + Sec.	2.48	379	0.47	4.3	0.25^e^		0.10	0.31
TEPA-Et-PMO^49^	TEPA/Prim. + Sec.	3.04	530	0.56	4.7	0.29^e^		0.10	0.66
PhPMO^48^	—	**—**	1004	0.69	2.5	0.37	25	—	0.55
NH_2_PhPMO^48^	Aniline/Prim.	1.85	924	0.70	2.4	0.40		0.22	0.37

Both grafting and organic bridge post-synthetic modification									
APTMS@NH_2_PhPMO^48^	Aniline + APTMS/Prim.	2.60	305	0.27	2.2	0.50	25	0.19	1.64

a Specific surface area.

b Pore volume.

c Pore diameter.

d Amine efficiency is calculated as the ratio between the CO_2_ adsorption capacity and amine loading. Under dry conditions, the maximum amine efficiency is 0.5. Under moisture conditions, the maximum amine efficiency is 1.0.

e Adsorption measurements performed using 5% CO_2_ gas flow in a TGapparatus; n.p. denotes for non-provided data; abbreviation nomenclature: TEOS (tetraethoxysilane); BTEPED (bis((triethoxysilyl)propyl)ethylenediamine); PE-MCM-41 (pore expanded MCM-41); AP and APTMS (3-(trimethoxysilyl)propylamine); ED, *N*-3 and TSPED (*N*-[3-(trimethoxysilyl)propyl]ethylenediamine); DT (*N*^1^-(3-trimethoxysilylpropyl)diethylenetriamine); 3-DEAPTES ([3-(diethylamino)propyl]trimethoxysilane); TMMAP ([trimethoxy[3-(methylamino)propyl]silane]); APTES (3-(triethoxysilyl)propylamine); TEIPS (triethoxy-3-(2-imidazolin-1-yl)-propylsilane); DAB (diaminobutane); DAH (diaminohexane); DADD (diaminododecane); DETA (diethylenetriamine); TEPA (tetra-ethylenepentamine); Prim. (primary amine); Sec. (secondary amine); Tert. (tertiary amine).

Our group has used this approach to modify SBA-15 with similar loading of amine moieties with distinct bulkiness such as primary [3-aminopropyltriethoxysilane (APTES)], secondary [trimethoxy[3-(methylamino)propyl]silane (TMMAP)], and tertiary ([3-(diethylamino)propyl]trimethoxysilane) (DEAPTES) amines and also with a diamine containing primary and secondary amine groups [*N*-[3-(trimethoxysilyl)propyl]-ethylenediamine (*N*-3)] (Fig. 2(a)). The impact of having silica surfaces anchored with such distinct amine moieties critically influences the CO_2_ adsorption capacity, gas selectivity and CO_2_ speciation as shown ahead.

Our research aims to study the influence of amine type (*e.g.*, primary, secondary) on CO_2_ adsorption^50^ and CO_2_/CH_4_ separation,^46^ the impact of water vapor upon CO_2_ adsorption on APTES- and DEAPTES-grafted SBA-15^51^ as well as the CO_2_-adducts formed in the amine grafted silica sorbents (see Section 3 for further details). The CO_2_ adsorption capacity increased from tertiary (DEAPTES@SBA-15), secondary (TMMAP@SBA-15), to primary amines (APTES@SBA-15), having the latter the highest ratio between CO_2_ adsorption and N loading (known as amine efficiency, ratio = 0.37, Table 1).^46^ The same amine efficiency for CO_2_ is obtained when the number of amines is almost 40% higher when both primary and secondary amines are used (*N*-3@SBA-15, Table 1). The selectivity towards CO_2_ in the CO_2_/CH_4_ mixture was higher in the presence of secondary amines (TMMAP@SBA-15 and *N*-3@SBA-15 sorbents, Fig. 2(a)). Similar conclusions can be perceived in other examples of AMPS^45^ listed in Table 1.

Alkyl amine-derived PMO (APTMS@PhPMO) obtained by the grafting method (Fig. 2(b)) seems to present a higher CO_2_ adsorption capacity when compared with similarly derived PMS (Table 1). APTMS@PhPMO shows a CO_2_ adsorption/N loading ratio twice the one obtained for the homologous SBA-15 material (APTES@SBA-15, Table 1), both tested at 25 °C.^48^ This enhancement of the adsorption capacity may be credited to the hydrophilic–hydrophobic character of the PMO sorbents, which can lead to a better homogeneity of the APTMS/APTES groups and a diffusion of the CO_2_ molecules along the pore channels. Nevertheless, it is essential to consider additional factors, including specific surface area, pore size, and pore volume, to ensure a more accurate and realistic comparison. Although this approach has provided materials with interesting properties, it comes with drawbacks such as low organic group loadings, which are often heterogeneously distributed within the pores, frequently inducing substantial pore volume reduction and channel blockage.

Our group also modified the Ph-PMO sorbent by an organic-bridge post-modification reaction through amination of the aromatic groups (NH_2_PhPMO) and by applying these two synthetic approaches (APTES@NH_2_PhPMO), Fig. 2(b), to study the influence of the type of amines on the CO_2_ adsorption–separation from CO_2_/CH_4_ gas mixtures.^48^ In tandem with computational modelling (*cf.* Section 5), we found that alkyl amines interact more strongly with the CO_2_ molecules than aromatic amines, such as the electron-donating pair of the N group is less available to react in the aromatic amines than in the case of alkyl amines. We also found that the type of amines is more relevant than their amount in the channels, as all these modifications lead to a decrease in amine efficiency compared with the APTES@PhPMO sorbent (Table 1).^48^

The organic-bridge post-modification (Fig. 1 and 2(c)) of PMO for CO_2_ adsorption with different proportions of primary and secondary amines and different chain lengths was also attempted by De Cank *et al.*^49^ The authors used a two-step approach to modify the ethylene bridge of Et-PMO with the alkylamines diaminobutane (DAB), diaminohexane (DAH), diaminododecane (DADD), diethylenetriamine (DETA) and tetra-ethylenepentamine (TEPA), Fig. 2(c). The authors found that this approach has advantages compared with the grafting procedure, such as higher thermal stability of the anchored amine groups. Additionally, they observed that the quantities of CO_2_ adsorbed are higher on PMO sorbents with low nitrogen content, showing the following CO_2_ adsorption capacity sequence PMO-DADD > PMO-DAH > PMO-DAB, which is the inverse of the modification degree (Table 1). A similar phenomenon is observed for the sorbents modified with tri- and penta-amines: PMO-DETA > PMO-TEPA.^49^

Developing the next-generation CO_2_ adsorbents relies on understanding how synthesis conditions impact the structural and textural properties of the sorbents, thereby influencing active adsorption sites at the atomic level. Achieving this requires the preparation of materials with control over the quantity and homogeneity of surface amine coverage. This aspect often implies performing grafting of organic moieties at a controllable intermolecular distance while maintaining the mesostructure. This approach addresses fundamental questions such as unravelling the role of silanol groups in CO_2_ speciation and uptake, identifying the optimal type of amine functional groups and the necessary degree of amine coverage for maximizing CO_2_ adsorption/selectivity. Moreover, it also helps to obtain atomic-level evidence about the benefits of cooperation between neighboring functional groups in CO_2_ speciation/uptake through a combination of ssNMR and computational methods. These methods allow structural elucidation of the CO_2_ species formed at confined surfaces at atomic resolution to be obtained. However, the bottleneck for future ground-breaking atomic level structural details on the CO_2_ adsorption mechanisms lies in the development of novel surface modification strategies targeted to boost the capabilities offered by NMR spectroscopy. The next section will provide insights on this subject.

### 2.3. Future perspectives in surface modification strategies towards the enhancement of NMR signal

**Fig. 3:**
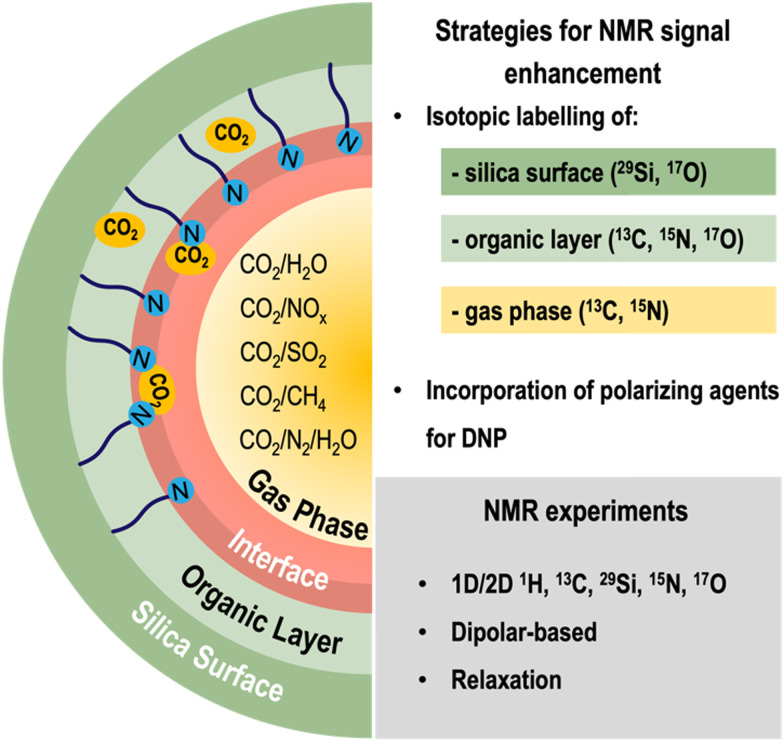
Overview of the methodology used for the ssNMR characterization of gas–solid interactions in silica CO_2_ adsorbents, involving strategies to enhance the NMR signal (namely isotopic labelling of the silica surface, the organic layer and/or the gas phase, as well as the incorporation of polarizing agents for MAS-DNP studies) as well as the main NMR experiments that may be explored for this application.

Although ssNMR is a powerful technique for studying the structure and dynamics of silica CO_2_-sorbents, it is often hampered by its inherently low sensitivity. Several strategies are being explored to overcome such limitations: (i) improvements in hardware (*e.g.*, high-field magnets) and pulse sequences, (ii) isotope labelling (such as ^13^C and ^15^N), and (iii) use of polarization techniques such as dynamic nuclear polarization (DNP), which consists in transferring polarization of electrons (located in polarizing agents) to the target nuclei (Fig. 3). Regarding (ii) and (iii), both isotopically labelled molecules and the polarizing agents may be incorporated during the synthesis and/or post-modification of silica materials, an activity that is currently being explored by many researchers, including our group.

Isotope labelling involves exchanging some or all the naturally occurring isotopes of a compound with NMR-active isotopes (*e.g.*, ^2^H, ^13^C, ^15^N, *etc.*). When studying CO_2_ adsorption, some dilute chemisorbed CO_2_ species are hard to detect due to lack of sensitivity; hence, to overcome this limitation, our group resorted to the use of ^13^C-labelled CO_2_ (^13^CO_2_) loaded materials to study CO_2_ speciation in AMPS.

Using this approach, it was possible to obtain new information on the nature of CO_2_ chemisorbed species in AMPS sorbents,^50^ reinforcing the potential of other isotope labelling schemes such as ^15^N and ^19^F into the organic moieties of porous adsorbents. For instance, introducing fluorine atoms may not only confer improved chemical stability to the sorbent^52–54^ but also significantly enhance CO_2_ adsorption capacity and selectivity in CO_2_/N_2_ gas mixtures, while maintaining the NMR sensitivity conferred by the high natural abundance of ^19^F isotopes.^55^ The replacement of hydrogen with fluorine atoms also enhances the hydrophobicity of the materials rendering this choice more attractive for applications under moist conditions where water competes with other gases for the same adsorption sites.

Combining ^13^C and ^17^O isotope enrichment of the CO_2_ molecule may potentially enable the unambiguous assignment of CO_2_ species that are very challenging to assign using ^13^C alone as their chemical shifts fall in a very narrow spectral region. However, the potential of ^17^O NMR in this field might be hindered by the prohibitively high cost of ^17^O-labeled CO_2_ (*ca.* 10 000 € per L when available). More affordable paths for isotopic labelling of gases are key to get the most of ssNMR applied to gas-loaded sorbents.

The other strategy used to enhance NMR sensitivity results from the application of DNP methods, profiting from the higher polarization of unpaired electron spins compared to the nuclear spins under the same experimental conditions. However, the process of introducing the polarizing agent, while maintaining the temperature and pressure conditions necessary for the gas adsorption is an enormous challenge and requires dedicated strategies. Traditionally, the polarizing agents, usually organic radicals, are introduced in the system^56^ either through impregnation methods^56,57^ or by grafting and co-condensation.^58–60^ These methodologies must be adapted for the case of gas adsorption studies. For instance, new radical formulations customized for porous materials and/or impregnation protocols under controlled conditions of temperature, pressure and gas composition, need to be developed to target this relevant niche application.

## 3. Studying CO_2_ adsorption mechanisms combining NMR and modelling

### 3.1. CO_2_ speciation studies

NMR spectroscopy has been widely employed to probe the local structure and dynamics of both the host framework and adsorbed guest molecules within porous materials.^28,61–68^ In the last few years, ssNMR has become the technique of choice to investigate the nature of CO_2_ species formed in AMPS materials, mostly under dry conditions.^50,51,61,63,69–74^ Traditionally, CO_2_ speciation studies arising from chemisorption exploit chemical shift (CS) analysis that can be performed with accuracy in combination with computer modelling. However, recently, NMR relaxometry in combination with chemical shift anisotropy (CSA) analysis has been explored to elucidate the speciation of the physisorbed fraction based on distinct dynamic behaviour.^73,74^ In the following section, the most recent and impactful works will be briefly discussed.

**Fig. 4:**
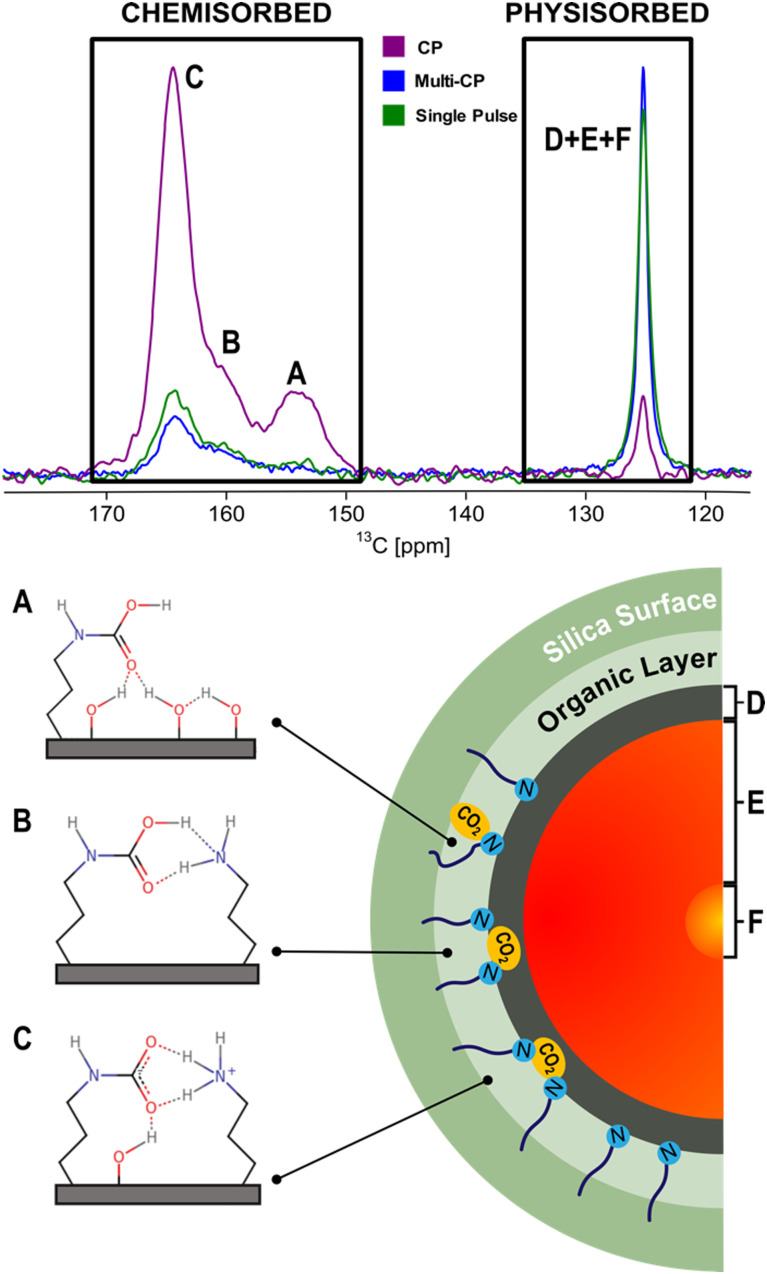
(top) Comparison between the ^13^C CPMAS (purple), MultiCP (blue) and single pulse (green) NMR spectra of dry APTES@SBA-15 after ^13^CO_2_ adsorption (bottom, left). Scheme of the three hypothesised CO_2_ chemisorbed species (A)–(C) formed in APTES@SBA-15 loaded with ^13^CO_2_ (*P* = 770 torr) as described in previous studies.^50,71^ (bottom, right) Scheme of a simplified CO_2_-filled pore of APTES@SBA-15 after adsorption, containing the physisorbed species (D)–(F) shown in our recent work.^73^Fig. 7 will further detail how the physisorbed CO_2_ peak is decomposed into three distinct components. Adapted from ref. 73 with permission from ACS, copyright 2021.

In a pioneering NMR study that included computer modelling, ^13^C NMR resonances were assigned for the first time to specific structures of chemisorbed CO_2_ species on SBA-15 functionalized with propylamine groups, exposed to variable CO_2_ pressures (from 1.3 mbar to 1 bar) under dry conditions.^69^ Three distinct chemisorbed CO_2_ species were identified with different populations determined by the CO_2_ partial pressure employed (details in the description of the apparatus utilized for variable-pressure ssNMR below). The species include two variants of carbamic acid species (A, B) and one alkylammonium carbamate ion pair (C) (Fig. 4). Note that among the three ^13^C ssNMR methods used, only the MAS and MultiCP spectra offer quantitative results, with the latter delivering about an 18-fold reduction in experimental time.

The successful completion of this study relied heavily on the precise control over key variables, namely temperature, partial pressure, and humidity.

To regulate these conditions, an experimental setup was constructed (Fig. 6), consisting of a custom-built high-vacuum apparatus linked to a turbomolecular pumping station capable of achieving a vacuum level greater than 10^−4^ Pa. A borosilicate glass container was connected to the vacuum apparatus and utilized as a chamber to house an NMR rotor. This arrangement facilitated the degassing and heating of zirconia NMR rotors under high vacuum conditions, with temperatures reaching up to 300 °C. The heating process was carried out using a specifically designed oven, which was connected to a power controller, and the temperature was monitored using a thermocouple. The desired gas was introduced into the system through a canister connected to both the vacuum apparatus and the glass container. The pressure within the glass container was measured using a capacitance transducer. Subsequently, our innovative sorption device served as inspiration for further research on CO_2_ adsorption on amine-functionalized MOFs utilizing ssNMR spectroscopy.^75^

In 2018, T. Čendak *et al.* helped to elucidate how the proximity of amine groups affects the formed CO_2_ species in SBA-15 silica, using ^13^C CSA NMR experiments.^50^ Two strategies were employed to modify the average distance between the anchored amine groups. First, the amine surface density was varied by changing the quantity of loaded amine groups, and second, amine moieties were protected with bulky carbamate groups ((3-triethoxysilylpropyl)-*tert*-butylcarbamate: TESPtBC) to prevent the clustering of the propylamines during grafting. The TESPtBC group is easily removed by heat prior to CO_2_ adsorption, thus regenerating the initial primary amines with augmented inter-amine spacing. This second approach favoured the formation of isolated propylcarbamic acid species, while the first promoted ion pair species due to the formation of amine aggregates. To determine the protonation state of each chemisorbed CO_2_ species, we monitored the changes in the principal values of their ^13^C CSA tensors by means of spinning sideband analysis through ^13^C cross-polarization (CP) NMR, at low MAS rates (2 kHz). A comparative evaluation of our results *versus* a repository of ^13^C CSA tensor parameters comprising 70 protonated and deprotonated carboxylates found in amino acids, facilitated the successful assignment of resonances corresponding to either aggregated or isolated CO_2_ species.^50^ In addition, Shimon *et al.*^76^ examined the system of a propylamine-functionalized SBA-15 when exposed to CO_2_. The material was loaded with 1 bar of CO_2_ and ^15^N CPMAS NMR experiments were carried out, with both carbamic acid and carbamate being observed in the results. Although CP is not quantitative, it was concluded that both carbamic acid and carbamate were formed after exposure to CO_2_, with the former desorbing faster than the latter. The authors also noted that, 100 hours after loading the sample with CO_2_, there was less carbamic acid than initially, while the quantity of carbamate remained constant.

While chemisorbed species often exhibit distinctive ^13^C CS, the physisorbed CO_2_ shows a single resonance at 125 ppm (Fig. 4). Our group showed that it is possible to discriminate among three physisorbed CO_2_ species using ^13^C NMR relaxation and ^13^C CSA measurements.^73^ The ^13^C MultiCP spectrum of CO_2_@APTES-SBA15 displays the presence of spinning sidebands, which can be associated with a fraction of CO_2_ molecules (species D) with fewer degrees of freedom when confined. Deconvolution analysis demonstrates that the signal with spinning sidebands corresponds to a small fraction of the full physisorbed CO_2_ (15%). Moreover, relaxation studies, namely *T*_1_ measurements, showed three distinct superimposed components associated with the single resonance of physisorbed CO_2_ at 125 ppm. These three CO_2_ species have *T*_1_ values of 2.4 s, 0.2 s and <500 μs, which fall in the range of CO_2_ species in a solid-like (species D), liquid-like (species E) and gas (species F) state, respectively (Fig. 4). The species D is associated with CO_2_ molecules directly interacting with the surface of the adsorbent, as confirmed by a ^1^H–^13^C HETCOR experiment,^73^ which clearly showed correlations between the ^13^C CO_2_ resonances and the ^1^H of the propylamine, and silanol groups from the silica surface. The distinct dynamics observed in species E and F could potentially be attributed to the density gradient spanning from the wall to the pore centre. This phenomenon may arise from the distance-dependent interaction between the CO_2_ molecules and the pore surface. Moreover, the combination of slow MAS/Multi-CP ^13^C NMR at MAS frequencies of 10 and 2.5 kHz and a *T*_1_ saturation-recovery experiment under quantitative conditions provides a toolbox for unambiguous identification and quantification of different CO_2_ chemi- and physisorbed species confined in AMPS. For the case of CO_2_@APTES-SBA15, the results showed that 45% of the total adsorbed CO_2_ was chemisorbed, with species A, B, and C representing 2%, 16%, and 27%, respectively. In the case of the physisorbed fraction, species D, E and F accounted for 8%, 39% and 8%, respectively. The methodology developed in this work is the basis for performing the NMR-assisted adsorption studies described in Section 4, also applicable to any other porous system.

Recently, chemisorbed CO_2_ species in MOFs and AMPS were studied by ^17^O ssNMR.^77^ The authors undertook a computational investigation to determine the utility of ^17^O ssNMR to probe CO_2_ capture chemistry. The simulations were supported by ^17^O NMR experiments on a series of MOFs loaded with C^17^O_2_, which indicated the formation of ammonium carbamate chains and a mixed carbamic acid–ammonium carbamate adsorption mode. The study was further extended to AMPS adsorbents: propylamine-SBA15 (Pr-Si) and triamine-SBA15 (Tri-Si) were loaded with 1083 mbar and 993 mbar of C^17^O_2_, respectively. The ^17^O MAS NMR spectra showed broad signals at 177.5 ppm and 28.7 ppm for Tri-Si, and 175.5 ppm and 33.3 ppm for Pr-Si. The signal at ∼175 ppm was assigned to ammonium carbamate oxygens, by comparison with Density Functional Theory (DFT) calculations for AMPS. However, the signals at *ca.* 30 ppm could not be assigned to any of the previously proposed carbamic acid, ammonium bicarbonate, or silylpropylcarbamate species. Therefore, the authors suggest either a new adsorption mode in AMPS, or isotopic enrichment of oxygen atoms in the silica backbone as possible explanations. This study highlights the utility of ^17^O NMR measurements for probing CO_2_ capture modes, opening the door to complement information retrieved from ^13^C NMR to study CO_2_ speciation in porous materials. However, as mentioned previously, the ^17^O enrichment of CO_2_ is prohibitively expensive.

### 3.2. Moisture-induced CO_2_ speciation

Water is a critical component of flue gases with varying proportions depending on the combustion source. The presence of water in flue gases has a major impact on the CO_2_ speciation formed. Hence, a careful investigation of the role of water is essential to optimise AMPS sorbents for CO_2_ capture in moist gas mixtures. Moreover, it has been shown that the presence of moisture prevents chemisorbed carbamic acid degradation into urea which leads to sorbent inactivation.^78^ Despite the importance of the role of water in the CO_2_ adsorption in silica porous materials, the subject has been weakly explored by NMR.^51,70,79,80^ This may be related to the difficulty of introducing an accurate proportion of water in the gas mixture.

**Fig. 5:**
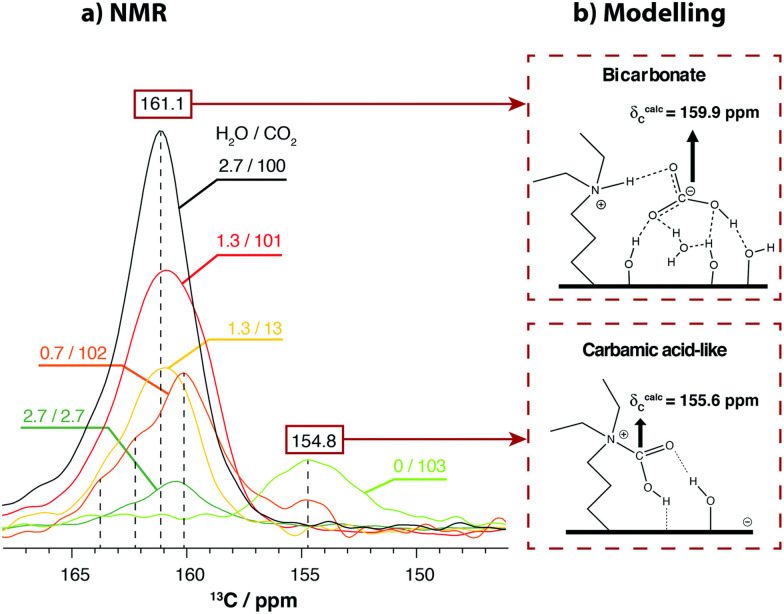
(a) ^13^C CPMAS NMR spectra of the chemisorbed species formed in DEAPTES@SBA-15 after exposure to different partial pressures of H_2_O followed by adsorption of ^13^CO_2_. Specific partial pressures are given in the figure, in kPa. The spectra were normalized with respect to the intensity of the propylamine carbons to facilitate comparison. (b) 2D representation of the optimised structures for bicarbonate and carbamic acid-like species. Reproduced from ref. 51 with permission from RSC, copyright 2021.

Amongst the few studies addressing the issue, our group investigated the CO_2_ speciation in SBA-15 derivatized with primary and tertiary amines in the presence of moist gas mixtures.^51^ The proportion of water was precisely controlled using a dedicated vacuum line. The study revealed that in the case of SBA-15 grafted with primary amines, although the presence of water does not affect CO_2_ capture, it promotes the conversion of carbamic acid into carbamate ion pairs by shuttling the proton from carbamic acid to a neighbour amine. The dominant CO_2_ species thus becomes the carbamate ion pair. In the case of tertiary amines, the formation of bicarbonate dominates the chemisorbed species with a small contribution from a carbamic acid species (Fig. 5). The experimental data were complemented by DFT calculations for the prediction of ^13^C CS for each species.

The CO_2_ speciation in SBA-15 grafted with tertiary amines has also been investigated by Chen *et al.*^80^ In this work, the water was introduced into the sample through the preparation of slurries resulting in samples with an uncontrolled proportion of water. The samples were exposed to pure ^13^CO_2_ and the resulting system was explored by ^13^C–^1^H HETCOR spectroscopy. They conclude that two types of bicarbonates were formed depending on two water environments. One resulting from the interaction with water in the proximity of silanol groups and another from the interaction with bulk water in the pore centre.

The CO_2_ speciation in deaminated silica has been recently addressed by Szego and co-workers.^79^ Chromatographic particles of porous silica Davisil LC60 were functionalized with a primary amine (APTES) and a diamine (3-(2-aminoethylamino)propyltriethoxysilane (AEAPTS)). Upon adsorption under both dry and wet conditions, the chemisorption of CO_2_ was analysed by a diversity of ssNMR methods. The single-pulse ^13^C and ^13^C CPMAS NMR spectra showed that the chemisorbed species in the dry samples were carbamic acids and carbamate ammonium ions, while in the wet samples, both species were detected together with HCO_3_^−^. The authors demonstrate that the CP efficiency to the HCO_3_^−^ was reduced as compared to the directly detected ^13^C NMR. However, ^13^C CPMAS NMR could still be used for qualitative assessment of the presence of HCO_3_^−^ without having to cool the samples down. This technique is a promising tool for evaluating the chemisorption of CO_2_ on mesoporous silica and could be useful for similar samples without isotopic ^13^C enrichment. Additionally, it was proposed that the presence of H_2_O in the aminated samples resulted in a sharpening of all signals (^1^H and ^13^C) under rapid MAS, suggesting an increased mobility of the species. This sharpening enabled the detection of two distinct types of carbamates formed upon CO_2_ chemisorption on the diaminated sample, as well as the resolution of most of the signals from the alkyl chains in the ^1^H domain. The high resolution of the ^1^H and ^13^C NMR spectra may provide a better understanding of how AMPS capture CO_2_ and promote their rational application in fields such as catalysis.

### 3.3. CO_2_ adsorption in gas mixtures

Understanding CO_2_ capture mechanisms in solid sorbents from realistic gas mixtures is an essential step towards the final application of these materials in real scenarios. Also, these studies are important when trying to find a particular application for a given solid porous material. For instance, the adsorption behaviour of a sorbent material will be strongly influenced by the origin of the gas mixture (flue gas, biogas, or direct air capture). In the last years, many studies have emerged focusing on the selective CO_2_ adsorption from real mixtures with different origins using distinct solid sorbents.^46,64,65,81–91^ However, few studies have been published investigating the adsorption mechanisms in AMPS by ssNMR. The studies published in this respect will be briefly discussed below, emphasizing the utility of NMR techniques to address the subject.

A pioneering work published in 2018 by our group^46^ demonstrated the potential of SBA-15 materials incorporating amine groups for CH_4_/CO_2_ separation, such as found in natural, bio and landfill gases. Aminosilanes of distinct steric hindrances were grafted onto SBA-15 silica and adsorption measurements were conducted up to 1000 kPa (10 bar) at 25 °C using CO_2_/CH_4_ mixtures, providing useful insights into potential applications. Remarkably, we observed extremely high CO_2_/CH_4_ selectivity with values of 1114 and 15 000 for secondary (TMMAP) and mixed primary/secondary (*N*-3) amine-functionalized SBA-15, respectively. This selectivity was attributed to the reaction of CO_2_ with surface amines, as confirmed by ssNMR of samples exposed to ^13^CO_2_. Furthermore, the primary and secondary amines remain unsaturated with CO_2_ at pressures below 40 kPa, suggesting that they can be partially regenerated by decreasing pressure. It was also reported that samples containing primary and secondary amines have a higher selectivity than SBA-15 due to their interactions with the polar carbamic acid species on the surface of the pores. Solid-state NMR data confirm this, as it reveals an interaction between the amines and CO_2_ not seen in SBA-15. At lower pressures, the relatively large mesopores and increased chemisorption of CO_2_ explain the higher selectivity, while polar interactions between the amines and CO_2_ further promote CO_2_ adsorption and decrease CH_4_ adsorption at higher pressures. Moreover, a preliminary evaluation of the materials' capacity for cyclic separation by pressure modulation revealed that TMMAP@SBA-15 and *N*-3@SBA-15 samples exhibit higher selectivity for CO_2_/CH_4_ separation, but lower working capacities than the primary amine APTES@SBA-15. The latter presents a better compromise between selectivity and working capacity, especially if a vacuum swing is used with a moderate vacuum (10 kPa regeneration pressure). Pacheco *et al.* in a follow-up work^81^ studied the influence of water in the separation of CO_2_/CH_4_ mixtures using AMPS materials. The adsorption behaviour of primary and tertiary AMPS (APTES@SBA-15 and DEAPTES@SBA-15) for CO_2_ and CH_4_ was studied under both dry and wet conditions. It was found that APTES@SBA-15 had a higher CO_2_ uptake than DEAPTES@SBA-15 due to the type and strength of interactions involving primary amines and CO_2_ at the pore surface. Meanwhile, DEAPTES@SBA-15 demonstrated an increase in CO_2_ uptake in the presence of pre-adsorbed water, attributed to the formation of bicarbonate species. This resulted in a much higher selectivity for CO_2_ in the CO_2_/CH_4_ separation for DEAPTES@SBA-15 than for APTES@SBA-15. Further investigations using ^13^C NMR spectroscopy and DFT calculations confirmed the formation of bicarbonate species when using tertiary amines under moist conditions, and a general stabilisation of the CO_2_ species formed when using primary amines. It was concluded that the low quantity of water present in a typical biogas stream would not significantly affect the CO_2_/CH_4_ separation in the first adsorption cycle but could lead to the observed effects during subsequent cycles. This work stresses the importance of the tandem use of DFT/ssNMR in addressing CO_2_ speciation in complex mixtures.

Flue gases discharged from coal-fired power plants contain not only carbon dioxide (CO_2_) but also acid–gas impurities, such as sulphur oxides (SO_*x*_) and nitrogen oxides (NO_*x*_), with typical concentrations ranging from 0.2 to 0.25 vol% and 0.15 to 0.2 vol%, respectively.^92^ The presence of these impurities poses various practical challenges in CO_2_ capture processes, leading to reduced adsorbent lifespan and capacity, adsorbent poisoning, decreased product purity, and increased operational expenses.^93,94^ While the existing literature predominantly focuses on the development and characterization of adsorbents with favourable CO_2_ capacities, limited attention has been devoted to the investigation of CO_2_ mixtures containing these acid-gas impurities. The lack of studies examining complex mixtures is predominantly attributed to the challenges arising from the corrosive and toxic nature of these gases. Razaei *et al.* examined the irreversible binding of SO_2_, NO, and NO_2_ to four supported amine adsorbents, assessing their adsorption capacities and their impact on CO_2_ adsorption capacities.^95^ The adsorbents consist of poly(ethyleneimine) and three silane coupling agents with primary, secondary, and tertiary amines, and their performance was evaluated using different characterization techniques, including ^13^C ssNMR. Under the experimental conditions used, it was observed that primary amines with high amine loadings exhibited a greater affinity for NO compared to their secondary and tertiary amine counterparts. However, the overall NO adsorption on the aminosilica adsorbents was found to be low, resulting in negligible changes in the CO_2_ capacities of the exposed adsorbent materials. Conversely, all materials displayed a notable adsorption capacity for nitrogen dioxide (NO_2_). Consequently, all adsorbents treated with NO_2_ demonstrated a substantial reduction in CO_2_ capacity, which can be attributed to the deactivation of amine groups caused by the irreversible binding of NO_2_. In materials with similar amine loadings, secondary amines exhibited a higher affinity for SO_2_; however, their loss in CO_2_ capacity after exposure to SO_2_ was lower than that of primary amines. This suggests that secondary amines are more stable in the presence of SO_2_, indicating greater desorption of SO_2_ from secondary amines during the desorption stage. These results highlight the need to reduce SO_2_ and NO_2_ concentrations in flue gas prior to the CO_2_ capture process. Conversely, the presence of NO does not significantly impact the capture efficiency of these materials. This suggests that such materials hold promise for post-combustion CO_2_ capture from flue gas streams derived from natural gas combustion, which typically exhibit reduced SO_2_ concentrations but significant NO_*x*_ concentrations. In a follow-up article, the same authors evaluate the stability of amine adsorbents to SO_2_ and NO_*x*_ in post-combustion CO_2_ capture by performing dynamic, multicomponent adsorption experiments in a fixed bed.^96^ This study investigates the effects of SO_2_, NO, and NO_2_ impurities on the dynamic adsorption capacity of amine-impregnated and amine-grafted silica adsorbents in CO_2_ adsorption breakthrough experiments where a defined mixture of gases flows through the packed-bed sorbent column. The experiments involve dual-component co-adsorption of SO_2_/CO_2_, NO/CO_2_, and NO_2_/CO_2_, as well as three-component SO_2_/NO/CO_2_ adsorption. The results indicate that SO_2_ significantly impacts the dynamic CO_2_ capacity of aminosilica adsorbents, but the adsorbents remain stable during co-adsorption if the bed is not fully saturated with SO_2_. NO shows little competitive effect on CO_2_ adsorption, attributed to the lower affinity of amine-based adsorbents towards NO compared to SO_2_. The presence of NO_2_ at low levels in the simulated flue gas has minimal impact on CO_2_ adsorption. Among the investigated adsorbents, those containing secondary amine groups demonstrate greater stability against SO_*x*_ and NO_*x*_ impurities in CO_2_ capture processes compared to adsorbents with primary amine groups. However, additional studies are required to address crucial aspects such as the potential adsorption mechanisms of these acid gases and the impact on the speciation of chemisorbed and physisorbed CO_2_ fractions. ssNMR spectroscopy will play a pivotal role in further investigations, as it possesses the capability to differentiate between distinct chemical and dynamic environments, regardless of the crystalline nature of the solid material.

### 3.4. Correlating CO_2_ structure and dynamics through NMR relaxation studies

Solid-state NMR has been used extensively to probe dynamics in biomolecular systems providing information about local interactions and the motional modes of molecular groups.^97–100^ In porous materials, studies of molecular motion have been performed in adsorbed CO_2_ in MOFs.^101^ However, NMR studies addressing the molecular dynamics of the different CO_2_ species in porous silica are scarce.^73,74,102–104^ The information obtained from these studies, far from being a scientific curiosity, is especially useful to assess important aspects such as stability or activation energies of the distinct CO_2_ species formed in confinement.

Recently, our group has conducted for the first time a comprehensive analysis of the molecular dynamics of chemi- (A, B and C) and physisorbed (D, E, and F) CO_2_ species in amine-modified SBA-15 using ssNMR to measure their rotating frame spin–lattice relaxation times (*T*_1*ρ*_).^74^ The chemisorbed species A, B, and C were found to have the shortest *T*_1*ρ*_ values (4.7, 1.1 and 1.4 ms, Table 2) as expected due to their highest rigidity. The *T*_1*ρ*_ value of 8.1 ms for species D, which is engaged in weak interactions with the silica surface, was found to be intermediate. For the two pure physisorbed CO_2_ species E and F, the *T*_1*ρ*_ values were much higher (10.0 and 44.0 ms, Table 2), reflecting their highly dynamic nature. Additionally, to obtain further insight into the dynamics of CO_2_ confined in amine-modified SBA-15, rotational correlation times (*τ*_c_) were estimated following the dependence of *T*_1*ρ*_ with the locking field (Table 2). The experimental data were fitted using either the Bloembergen–Purcell–Pound theory,^105^ for physisorbed CO_2_ species E, or the Bloch, Wangsness^106^ and Redfield theory^107^ for chemisorbed species A–D. The obtained *τ*_c_ values for species D (32 μs) and E (20 μs) are typical for molecular dynamics of a viscous liquid, while the chemisorbed species A–C exhibit longer *τ*_c_ values (162, 62, and 123 μs, respectively), reflecting the higher CO_2_ molecular rigidity. This analysis permitted the estimation of heteronuclear dipolar coupling constants and reduced CSA values, providing further insight into the nature of the adsorbed CO_2_. Previous studies combining ssNMR and DFT calculations^50,69^ coincide with these results, reinforcing the identification of the chemisorbed species A and B as two carbamic acid species, and C as an alkylammonium carbamate ion pair. These results further support the presence of at least two physisorbed species (D and E). However, the fast dynamics of species F made it impossible to carry out this analysis due to instrumental limitations.

**Table 2:** Relative fraction, longitudinal relaxation time (*T*_1_), rotating frame spin–lattice relaxation time (*T*_1*ρ*_), rotational correlation time (*τ*_c_), ^13^C–^1^H dipolar coupling constant (*b*_IS_) and reduced CSA (*δ*) for each CO_2_ species (A–F) adsorbed in APTES@SBA-15 after exposure to 770 torr of ^13^CO_2_ at room temperature

^13^CO_2_ species	Fraction%	*T*_1_/s	*T*_1*ρ*_/ms	*τ*_c_/μs	*b*_IS_/Hz	*δ*/ppm
A	2(1)	57(4)	4.7(1)	16(3) × 10^1^	69(3) × 10^2^	7(1) × 10^1^
B	16(5)	7(1)	1.1(1)	62(2)	153(3) × 10^2^	7(1) × 10^1^
C	27(8)	12.8(4 × 10^−15^)	1.4(1)	123(1)	22(1) × 10^3^	49(9)
D	8(1)	2.4(3)	8.1(1)	32(4)	40(2) × 10^2^	6(1) × 10^1^
E	39(1)	0.09(2)	9.4(9)	20(4)	—	0.20(3)
F	8(2)	<5 × 10^−4^	44(5)	—	—	—

## 4. NMR-assisted adsorption studies

### 4.1. Conventional approaches and their limitations

The performance of adsorbents for gas uptake is typically evaluated using manometric (also called volumetric) and gravimetric adsorption techniques. These methods provide a graph showing the partial gas pressure (or time) *versus* adsorbed gas amount at a constant temperature – an isotherm. The isotherm contains important information regarding sorbent's textural properties (specific surface area, pore volume, pore size distribution, *etc.*) and parameters related to its performance in gas capture (adsorption capacity, selectivity to a certain gas and isosteric enthalpy of adsorption, as the most important ones).^108^ However, despite their wide use, these conventional methods alone do not shed light on crucial questions such as, what is the contribution of the chemi- or physisorption to the adsorption process under these particular conditions? Neither do they give insight into the nature of adsorption sites. Therefore, to fully understand the performance of solids in gas adsorption applications, the use of additional techniques is mandatory. In this section, alternative techniques for elucidating adsorption properties of materials are discussed giving special attention to the ssNMR-assisted techniques.

### 4.2. Low-field-NMR-assisted gas “relaxorption”

Owing to the sensitivity to probe adsorbate–adsorbate and adsorbate–adsorbent interactions, NMR relaxation methods have been used to quantify adsorbed gases on solids enabling to register adsorption isotherms.^109–118^ In general, this approach covered a wide range of materials including zeolites, mesoporous silica, activated carbons and MOFs which were probed for the adsorption of CO_2_, CH_4_, C_2_H_6_, C_3_H_8_ and also vapours of MeOH, PrOH and H_2_O for gas capture/separation and other emerging applications. In most cases, these experiments were limited to the use of low-field magnets and carried out *ex situ*.^109–112,114^ In contrast, *in situ* tests were less common mainly due to the difficulties in designing and building the experimental setup. One alternative was found by integrating NMR with either a sorption analyser or a homemade gas dosing system.^115–119^ For instance, the adsorption of MeOH vapours was studied in the sorption apparatus coupled with the low-field NMR spectrometer providing simultaneous *in situ* and *in operando* measurements. Information on pore filling, structural heterogeneity and host phase transition was obtained.^115^ Furthermore, due to the unique properties of ^129^Xe (high sensitivity to the surrounding chemical environment resulting in a significant difference in CSs on ^129^Xe NMR spectra) it has been widely used as a probe molecule to study adsorbents.^120^ Its diffusion and adsorption on materials were followed to calculate specific surface area, pore volume, isosteric enthalpy of adsorption of gases on solid materials. To sum up, all these methodologies are limited to follow the physisorbed fraction of the adsorbate, mostly restricted to following ^1^H signals in the adsorbed molecule due to sensitivity reasons.

### 4.3. CO_2_ adsorption isotherms by high-field ssNMR-assisted techniques

**Fig. 6:**
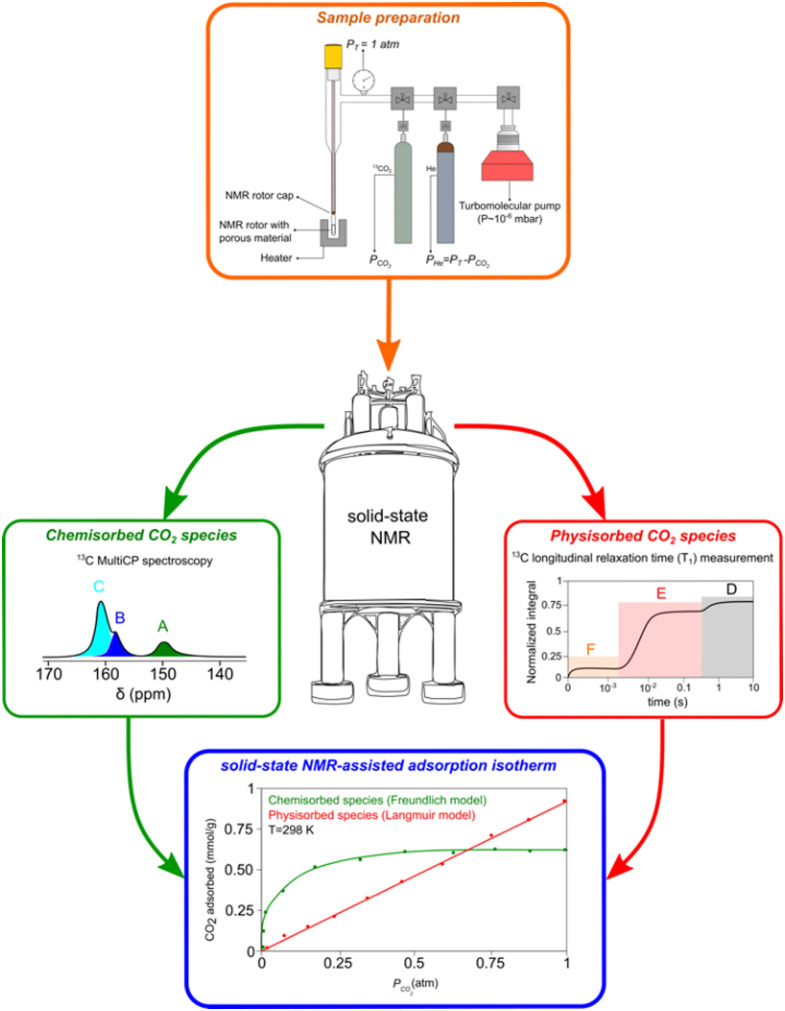
Schematics of the methodology used to perform qualitative and quantitative characterization of different chemi- (A)–(C) and physisorbed (D)–(F) CO_2_ species formed at different gas partial pressures. Reproduced from ref. 121 with permission from ACS, copyright 2023.

**Fig. 7:**
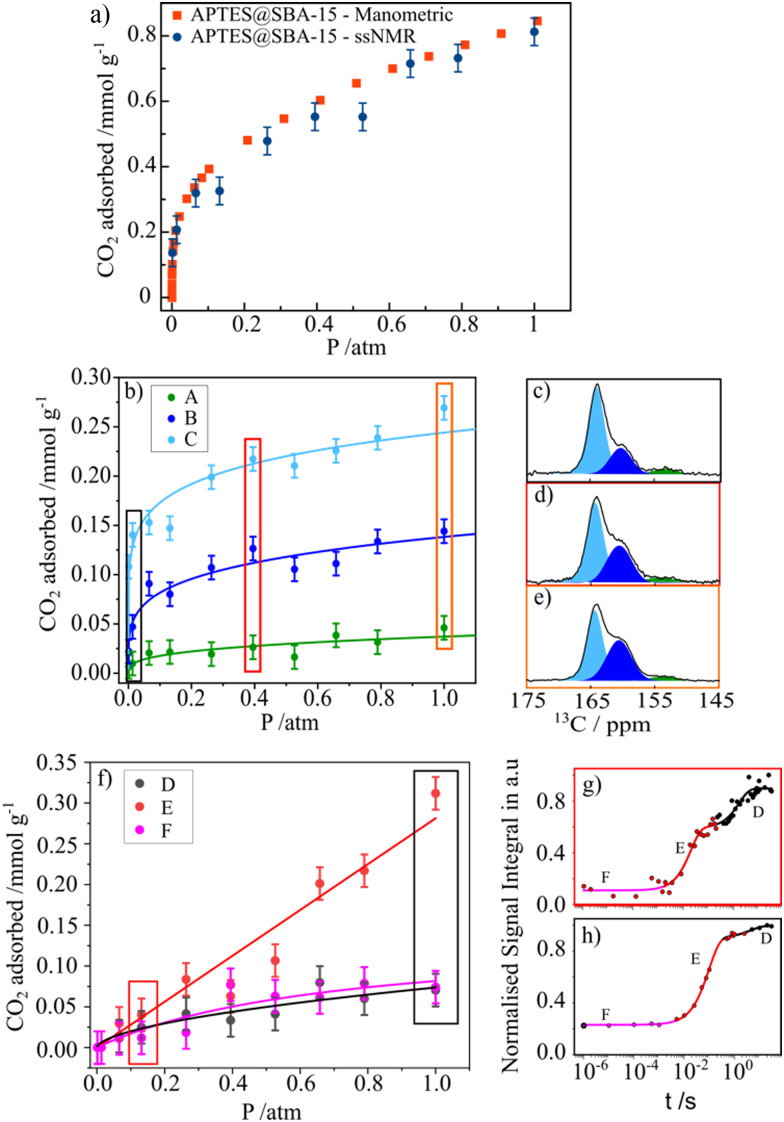
(a) CO_2_ adsorption isotherms (recorded at 298 K) for APTES@SBA-15 obtained by the manometric (red) and ssNMR (blue) techniques (vertical lines depict the error bars). (b) ssNMR isotherms of the chemisorbed CO_2_ components (A)–(C) fitted with the Freundlich model. (c)–(e) Selected ^13^C MAS NMR spectra corresponding to the isotherm data points recorded at 0.01, 0.39 and 1.00 atm, respectively. (f) ssNMR isotherm of the three physisorbed CO_2_ components (D)–(F) fitted using the Langmuir (E) and (F) and the Freundlich (D) models. (g) and (h) NMR saturation recovery curves of the corresponding isotherm data points recorded at 0.13 and 1.00 atm. Reproduced from ref. 121 with permission from ACS, copyright 2023.

As mentioned above, the use of NMR methods to collect adsorption data has been mainly limited to the use of low-field NMR. However, our group has tried to explore ssNMR methodologies to overcome these shortcomings as many advantages would arise from applying the standard high-resolution ssNMR features (high-field magnet and MAS), which are crucial to record full adsorption isotherms of confined non-protonated gas molecules. One of the first attempts to apply variable pressure ssNMR under MAS for the CO_2_ adsorption on mesoporous silica and detect different adsorbed gas adducts was made by our group.^69^ Moreover, in a follow-up work, it was demonstrated that the quantification of chemisorbed CO_2_ species is possible by using MultiCP NMR spectroscopy while the physisorbed species may be quantified by means of *T*_1_ measurements.^73^ Very recently, we have also shown that the ssNMR-assisted method is able to record full gas adsorption isotherms as it was reported for ^13^CO_2_ adsorption on APTES@SBA-15.^121^ The scheme with the different stages of our methodology is represented in Fig. 6. Recorded adsorption data were validated by comparison with the data obtained using conventional static manometric techniques (Fig. 7(a)).

Moreover, applying the ssNMR-assisted approach enabled the collection not only of the full adsorption isotherm, but also of individual isotherms for each chemi- (A, B and C, Fig. 7(b)–(e)) and physisorbed CO_2_ species (D, E and F, Fig. 7(f)–(h)), which is impossible using conventional adsorption isotherm methods. The obtained isotherms were fitted using Langmuir, Freundlich or Sips models and corresponding parameters (maximum gas uptake, affinity of the gas to the material and heterogeneity of the adsorption sites) were derived from them. This methodology is already being applied by our group to a broader list of materials such as zeolites, carbons and MOFs, thus showing its potential in tailoring more efficient materials for gas adsorption and separation processes.

## 5. Computational modelling of silica-based sorbents at the meso- and molecular-scale

Computational studies provide insights into the gas–surface interactions in a myriad of materials, including silica-based sorbents. Computational tools have already been applied to (i) assist ssNMR to unravel the mechanisms of CO_2_ adsorption on AMPS,^46,69,71^ (ii) understand and predict the CO_2_ physisorption performance of a variety of PMO materials displaying different chemical functionalities^48,122–125^ and (iii) simulate dynamic gas sorption measurements on silica-based sorbents.^126–129^ Apart from silicas, these studies place significant emphasis on investigating other materials such as MOFs, COFs, and zeolites.^130,131^

### 5.1. Modelling silica-based adsorbents

#### 5.1.1. Crystal-like silica models

**Fig. 8:**
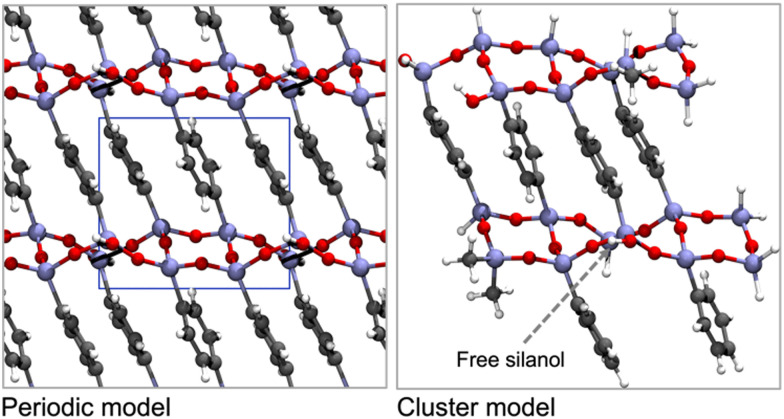
Top views of the periodic model (left, with the unit cell depicted inline) and the selected cluster model (right) for Ph-PMO. The colour code shows H in white, C in grey, O in red, and Si in beige. Schemes are based on the models presented by Lourenço *et al.*^123^

The intercalation of organic/inorganic groups into the pore walls of crystalline-like materials, such as PMO, is more attractive for computational studies due to the easiness in modelling their structures, thereby rendering them suitable for the generation of both periodic^46,116,118^ and cluster models^123,125^ of the surface PMO walls. Two types of periodic models of PhPMO material were constructed for the first time by Martinez and Pacchioni^122^ to study the interaction between their surface and CO_2_ and CH_4_ molecules. The use of a periodic supercell (Fig. 8) allowed the simulation of simultaneously both the long-range order of the lattice and the short-range nature of the interaction with gas-phase molecules. The influence of defects, impurity atoms and alterations in the organic and inorganic parts of the lattice on the adsorption properties of the sorbents were also studied in this work.

**Fig. 9:**
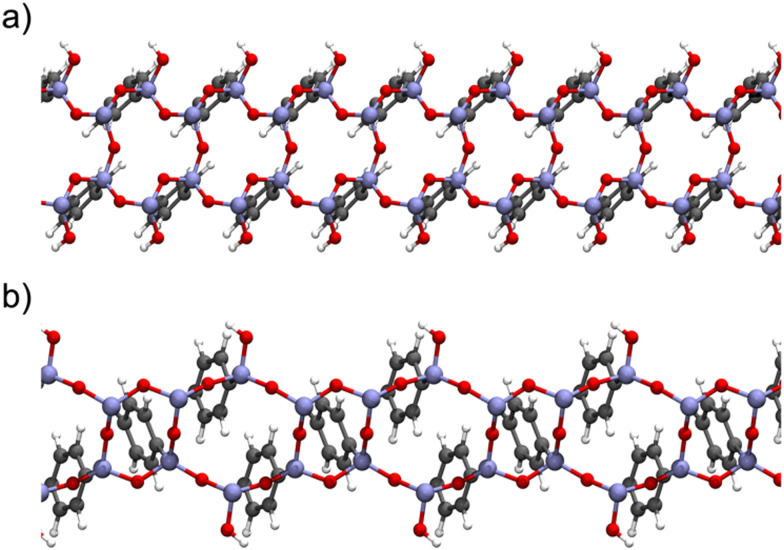
Lateral view of the Ph-PMO periodic models (a) encompassing six- and four-member rings of organosilica with T^3^ to T^2^ silicon environments in a 2 : 1 ratio and (b) corresponding to a regular sequence of six-member silica ring with alternate T^3^ and T^2^ species in a 1 : 1 ratio. Schemes are based on the models presented by Martinez and Pacchioni.^122^

Later, our group selected a model encompassing six- and four-member rings (Fig. 9) of organosilica with T^3^ to T^2^ silicon environments in a 2 : 1 ratio (T^*n*^ = RSi(OH)_(3−*n*)_(OSi)_*n*_, R represents the organic bridge), as this seemed more realistic considering the characterization data from ssNMR obtained by Comotti *et al.*,^132^ for studying the interaction of gas molecules on the surface of a variety of PMO sorbents.

Cluster models are more affordable than periodic models, and their validation is fundamental to the computational study of the interaction of different gas molecules at the surface of PMO structures. Our group observed that both types of models,^48,125^ were accurate in the determination of CO_2_ and CH_4_ adsorption performances on Ph-PMO sorbents, as the obtained data agrees with the experimental adsorption measurements, regardless of the chosen DFT exchange–correlation functional (Table 3 and Fig. 8).

**Table 3:** Energies and CO_2_/CH_4_ selectivity for CO_2_ and CH_4_ adsorption in the periodic and cluster models of Ph-PMO calculated using different methods

Gas	Model^a^	DFT method	*E*_ads_ (kJ mol^−1^)	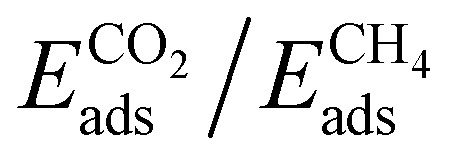 b ratio
CO_2_	Periodic PW/PAW	PBE-D2	−21.7	2.24
	Cluster GTO	PBE-D2	−25.8	1.79
		M06-2X	−25.1	2.46
	Experimental^c^^d^		−19	3.2^e^
CH_4_	Periodic PW/PAW	PBE-D2	−13.1	
			−10.1^f^	
	Cluster GTO	PBE-D2	−14.4	
		M06-2X	−10.2	
	Experimental^c^^g^		−12.6 ± 0.8	

a PW/PAW and GTO stand for plane-wave/projected-augmented wave and Gaussian type orbitals, respectively.

b Ratio between the calculated adsorption energies of CO_2_ and CH_4_.

c Experimental isosteric heat of adsorption.

d From adsorption isotherms (temperatures, pressure range and uncertainty not specified) in ref. 132.

e Ratio between the Henry's constant for CO_2_ and for CH_4_ determined in ref. 48.

f Second most stable structure for the PBE-D2 method.

g From adsorption isotherms at *T* = 285 K, 298 K and 314 K and pressures of 0.01 to 0.1 MPa determined in ref. 133.

Although the cluster model represents a small fraction of the silica wall surface structure, its validation allows studying the adsorption of diatomic (*e.g.*, CO, H_2_, N_2_, O_2_, and NO), triatomic (*e.g.*, CO_2_, H_2_O, H_2_S, and SO_2_) and tetratomic (SO_3_ and NH_3_) gas molecules onto the PhPMO pore wall surface at lower computational cost compared with the analogous periodic system.^123^ The CO_2_ and CH_4_ adsorption behaviour of other PMO sorbents containing different organic moieties, such as biphenylene (Bph-), pyridine (Py-) and bipyridine (Bpy-) bridges and their mixtures (Ph/Py- and Bph/Bpy-moieties), was also assessed in previous studies.^125^ Building these cluster models presents a significant challenge, requiring consideration of the molecular-scale periodicity determined by XRD analysis of experimentally prepared PMO materials. The periodicity varies depending on the size of the target organic moiety. A convenient approach to build the models involves using the existing and optimized Ph-PMO model as a starting point. The next step is to replace the phenylene moieties of the material with desired organic bridges while considering its molecular-scale periodicity, followed by geometry optimization (DFT energy minimisation).^125^

#### 5.1.2. Amorphous silica models

The major challenge in exploring computational tools to study amorphous silica sorbents lies in building realistic atomistic models of their porous structures. As opposed to MOFs, that contain a well-defined structure, the amorphous structure of a silica is difficult to model due to its large degree of randomness.

**Fig. 10:**
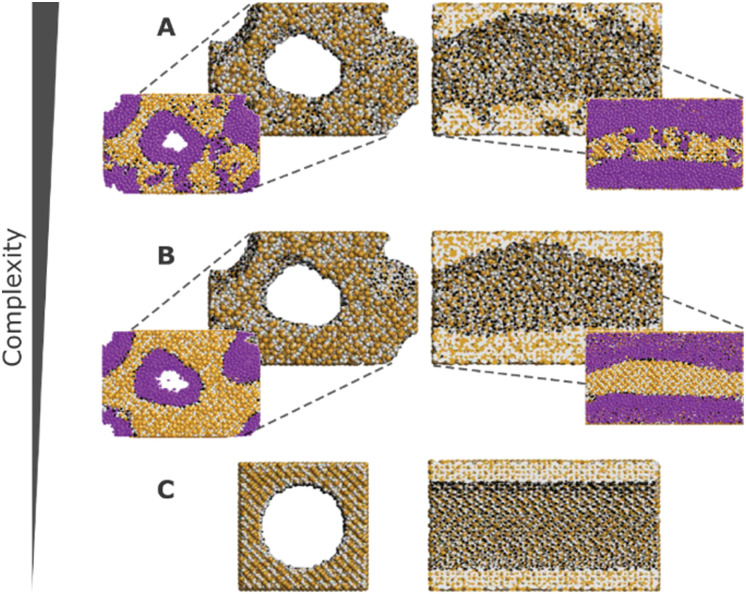
Front and cross section views of different models with decreasing complexity. Silicon, oxygen, and hydrogen atoms are represented in tan, grey, and black, respectively. Argon (represented in purple) adsorption snapshots for a relative pressure of *P*/*P*_0_ = 0.67 are also depicted for models A and B. Model A possesses meso- and microporosity, while model B exhibits no microporosity, and model C is a simplified cylindrical pore model. Adapted from ref. 126 with permission from ACS, copyright 2009.

These challenges have, over the years, been tackled with the development of more realistic models for mesoporous silicas, ranging from reconstruction to mimetic models. The former models are custom-designed to replicate structural properties observed in experimental studies, while the latter models aim at simulating the material synthesis process using molecular simulation techniques (Fig. 10).^126^

In computer simulations, the complexity of the system must be tailored to suit each specific problem, including the computational cost. This can range from more realistic structural models that incorporate both mesopores and micropores with appropriate pore roughness, to simpler cylindrical pores created from crystalline or pre-amorphized silica blocks. The modelling of such systems can be accomplished through multiple pre-processing approaches, including both specialized software and programming-based approaches. For instance, widely recognized molecular builders like Moltemplate and PACKMOL, although primarily used for biological and soft-condensed matter systems, can also be adapted for modelling silica materials. While these tools were originally developed for different applications, they offer versatile functionalities that can be harnessed to construct and arrange silica structures in a systematic manner. Another valuable tool for molecular modelling of silica systems is VMD^134^ (Visual Molecular Dynamics). VMD packages, like InorganicBuilder, offer user-friendly interfaces and tools specifically designed for modelling inorganic materials. These packages provide a convenient way to construct and manipulate silica structures, making them accessible to researchers with varying levels of expertise. Alternatively, for more advanced modelling and simulation techniques, researchers can leverage their programming skills. They can opt to use programming languages like Python and take advantage of specialized packages such as PoreMS^135^ or MoSDeF (Molecular Simulation Design Framework),^136^ which provide functionalities tailored to this type of systems. These programming-based approaches offer greater flexibility and customization options, enabling the implementation of sophisticated algorithms and to perform complex simulations tailored to specific research objectives.

In certain instances, the utilization of a complex model becomes necessary to ensure the accurate simulation of the desired properties. Recognizing this, for a more precise and comprehensive modelling of the system, it is crucial to employ a synergistic combination of various molecular simulation techniques. By deepening our understanding and effectively integrating these techniques, we can achieve a more refined representation of the system, enabling us to capture intricate details and accurately predict the properties of interest. Bhattacharya *et al.* used coarse-grained simulations of surfactants to obtain a model of the main physical features of the pore.^126^ It is noteworthy that the mentioned methodologies are most widely described in the literature, as some novel modelling methods like self-assembly of silica-based mesoporous materials or modelling materials using reactive force fields are starting to emerge.^137^

A proper characterization of the model must be performed, to confirm its validity. This can be achieved by comparing structural properties like the pore size distribution, specific surface area, framework density, surface hydroxyl group density, and many others, with experimental values. In contrast to periodic crystalline materials, these silica models must also ensure a suitable degree of amorphization, which can be obtained using quenching methods like temperature increase through molecular dynamics canonical ensemble simulation.^138^ Recently, machine-learning driven molecular dynamics has also been employed in the simulation of accurate amorphous silicon materials.^137^

The simulation of gas adsorption solely on bare silica surfaces has limited relevance. As a result, these materials are predominantly simulated as a support medium for organic molecules, particularly amines. Hence, the models undergo subsequent functionalization procedures prior to gas adsorption studies, as described in the following section.

#### 5.1.3. *In silico* functionalization strategie**s**

Post-functionalization is another big challenge in the construction of realistic models, as the distribution degree of functional groups on the porous material is unknown. The heterogeneity of the surface coverage depends on several factors associated with the functionalization precursor (*e.g.*, concentration, size, kinetics, *etc.*), adsorbents (*e.g.*, pore size and volume, particle size, number and type of available silanols, *etc.*) and synthesis conditions (solvents, reaction time and temperature, *etc.*) as discussed in Section 2. Moreover, the large number of available functionalization precursors is also a drawback for selecting the most suitable adsorbent for gas adsorption–separation applications as hundreds of silica-based sorbents may be produced and experimentally testing all of them becomes impracticable.

Computational tools are thus an environmentally friendly way to predict if different sorbents could perform better without the need to synthesize them in the lab. Up to now, to design the functionalized sorbent models, the desired functional group replaces a selected hydrogen atom. Typically, this is made on all possible functionalization sites, as the distribution of the functional group on the material is mostly unknown, generating several geometries that must be optimized and studied. Our group performed this type of model building on both cluster PMS^46,69,71^ and periodic PMO models.^48,124^ In the case of PMO sorbents, functionalization is easier to predict, as the crystal-like structure and fewer possible defects are an advantage in analysing data from characterization techniques like PXRD, elemental analysis and ssNMR.^124,125^

**Fig. 11:**
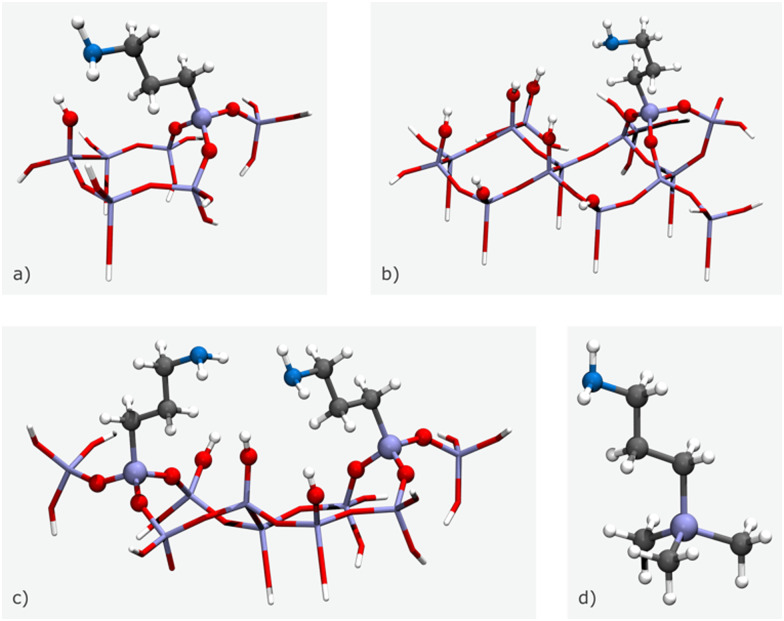
3D illustrations of the different size clusters: (a) small cluster, (b) medium cluster, (c) large cluster, and (d) isolated amine. Stick and ball-and-stick representations indicate frozen and fully optimised atoms, respectively. The colour code is as follows: white for H, dark grey for C, blue for N, red for O, and purple for Si. The schemes are based on the models presented by Mafra *et al.*^69^

However, for modelling specific structures, certain structural parameters may need to be fixed at non-optimal values, which can result in vibrational modes with associated imaginary wavenumbers. Cluster size can vary depending on the number of amine chains and surface silanols, with small clusters composed of a single amine chain and surface silanol (Fig. 11(a)), medium clusters with two amine chains and a surface silanol (Fig. 11(b)), and large clusters made up of one amine chain and five surface silanols (Fig. 11(c)). For the case of an isolated amine, a (CH_3_)_3_SiCH_2_CH_2_CH_2_NH_2_ model can be applied, as it is unable to form hydrogen bonds with the silica framework (Fig. 11(d)).^71^ In this model, terminal OH moieties are replaced by non-interacting methyl groups. While functionalization tasks have traditionally been accomplished through manual manipulation of the model, the advent of software packages mentioned in the previous section has significantly simplified this process. These software packages provide a user-friendly interface that enables the swift and convenient introduction of different functional groups in varying proportions, facilitating easy customization of the models.

For instance, to graft silylpropylamines onto the clusters, optimization is employed to bind three surface OH groups by each alkylamine. Subsequent optimizations involve the relaxation of various species, such as the alkyl chain (along with its respective functional group at the end), water or CO_2_ molecules (when present), the SiO_3_ moieties binding the alkylamines, and the surface OH groups.^71^ The remaining Si and O atoms are held constant at their crystallographic positions during these optimizations. The absence of imaginary values in the phonon mode frequencies pertaining to the atoms optimized in the various structural models confirms that these structures are true minima on their potential energy surfaces.

Additionally, for constructing more intricate functionalized models, pre-processing simulations can be employed. Techniques like Configurational Bias Monte Carlo (CBMC) offer a controlled approach to grow chains, enhancing the precision of surface functionalization. These methods allow researchers to achieve a more refined and tailored functionalization of the surface, expanding the possibilities for studying complex systems in depth.^139^

### 5.2. Modelling NMR parameters

The development of software dedicated to modelling ssNMR parameters has gained significant traction in recent years. Prominent among these software packages are SIMPSON, SpinDynamica, SPINEVOLUTION, Spinach, and EXPRESS. These platforms offer a versatile suite of simulation capabilities, empowering users to perform different experiments across various spin systems within a virtual NMR spectrometer.^28^

In the context of investigating the complexities inherent to porous adsorbent materials, researchers frequently integrate ssNMR data with theoretical methodologies.^50,71^ Central to this synergistic strategy is the combination of NMR lineshape simulations, DFT calculations, and molecular dynamics (MD) simulations, collectively fostering a holistic comprehension of these materials.^71,140^

DFT calculations, which are rooted in the fundamental principles of electronic structure determination, can be used to compute tensors related to various NMR phenomena, such as CS, quadrupolar coupling, hyperfine interactions, and binding energies. These tensors can then be compared with experimental data to provide insights into the nature of adsorption sites.

### 5.3. Modelling pore surface interactions with adsorbates

#### 5.3.1. CO_2_ chemisorption and physisorption

Computational investigations of CO_2_ chemisorption in porous materials are primarily centred on exploring the formation of covalent bonds between CO_2_ molecules and the material. Specialized techniques like *ab initio* molecular dynamics (AIMD) and DFT calculations are widely employed to delve into this process. AIMD simulations enable the dynamic exploration of reaction pathways, energy barriers, and intermediate states during CO_2_ chemisorption.^141^ On the other hand, DFT calculations provide valuable insights into the electronic structure, energetics, and stability of CO_2_ adsorption complexes in a more reasonable timescale. While AIMD and DFT accurately capture quantum mechanical effects, they are computationally demanding, limiting the system size and timescales that can be studied. Reactive force fields offer more efficient simulations, allowing the study of larger systems and longer timescales. However, they rely on empirical potentials, which might not fully capture quantum effects or accurately describe complex systems.^142^ Recent advancements in machine learning potentials (MLPs) have revolutionized computational materials science. MLP models make use of techniques like neural networks and Gaussian process regression to provide highly efficient and accurate descriptions of potential energy surfaces.^143^ By leveraging these diverse computational approaches, researchers can attain a comprehensive understanding of the intricate mechanisms and thermodynamics associated with CO_2_ chemisorption in porous materials.

In addition to the chemisorption process, CO_2_ molecules can also interact with the amine functionalities without establishing chemical bond(s). In this case, the interaction between the adsorbate molecules and the surface of the material occurs by a physisorption process mediated by van der Waals forces.

Our group studied both processes by combining DFT and ssNMR studies.^46,71^ DFT studies were relevant to distinguish physi- and chemisorption by simulating the CO_2_ physisorption on the surface of three amine-modified silica sorbents (SBA-15 modified with APTES, TMMAP, and *N*-3) and comparing the experimental ^13^C and ^15^N NMR CS with those obtained computationally (see Section 3 for further details). This approach was also used to verify that physisorbed CO_2_ can interact with the chemisorbed species, giving a possible explanation for the high CO_2_ adsorption capacity and selectivity (on CO_2_/CH_4_ gas mixtures) observed even beyond the saturation of amines.^46^

In the case of PMO sorbents, DFT studies confirmed that CO_2_ is preferentially physisorbed on the PMO walls independently of the type of chemical functionalities introduced, being advantageous for adsorbent regeneration.^43,94–97^ The CO_2_ preferential adsorption on PMO materials without N groups, like Ph-^48,122–125^ and Bph-^125^PMO sorbents, occurs close to the isolated T^2^ type silanol species. The preferential adsorption sites seem to be the same on PMO sorbents with Py-, Bpy-, and Bph-moieties, or by mixtures of Ph/Py- and Bph/Bpy-species,^97^ but with the later sorbent showing enhanced CO_2_ adsorption.

The introduction of different types and amounts of amino groups on the PhPMO sorbent increased the CO_2_ adsorption energy (obtained from DFT calculations using periodic PMO models) and Henry constant for CO_2_ (
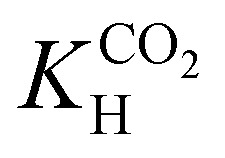
), showing a good agreement between calculated and experimental data.^48^ In this study, we also demonstrated that the modification of the PMO sorbents with alkyl amines (APTMS@PhPMO) improved the CO_2_ adsorption more than when aromatic amines (–NH_2_) were introduced into the material. Interestingly, the CO_2_ sorption affinity seemed to be more controlled by the amine type than by the nitrogen quantity in the PMO channels. Experimental and DFT studies displayed the same adsorption–separation trend, allowing the extension of the computational approach to study the CO_2_ adsorption–separation performance on other modified Ph-PMO sorbents, *e.g.*, containing –NO_2_, –NH-i-Pr, –CH_2_NH_2_, and –SO_3_H groups instead of aromatic amines. The PMO modified with CH_2_NH_2_ groups bonded to the phenylene moiety enhanced CO_2_ adsorption capacity.^48^

**Fig. 12:**
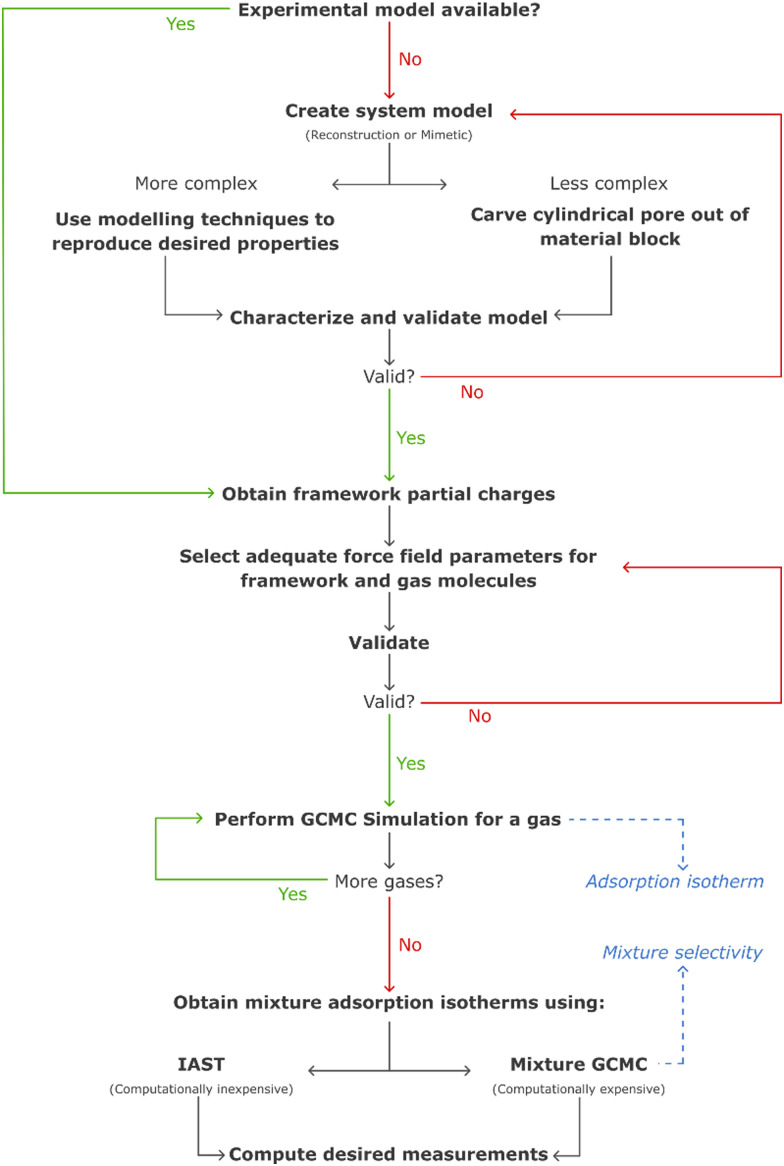
Flowchart illustrating a possible methodology used for gas adsorption simulations in porous frameworks (GCMC: grand canonical Monte Carlo; IAST: ideal adsorbed solution theory^147^).

Classical MD simulations are frequently used to model the motion and behaviour of CO_2_ molecules within porous materials, providing insights into diffusion, adsorption kinetics, and the dynamics of the sorption process.^144^ Another commonly utilized technique is grand canonical Monte Carlo (GCMC) simulation, which enables the prediction of adsorption isotherms and the determination of adsorption capacities at different temperatures and pressures, as depicted in Fig. 12.^144^ Advanced methods, such as replica exchange^145^ and umbrella sampling,^146^ can also be applied to explore the free energy landscape and overcome energy barriers associated with CO_2_ physisorption.

#### 5.3.2. CO_2_ sorption in gas mixtures

Recently, simulations of CO_2_ adsorption on gas mixtures that comprise N_2_, CH_4_, H_2_O, and many more have become increasingly popular as they are more reflective of real-world conditions, like those found in industrial and environmental processes such as carbon capture from flue gas and biogas purification. These studies delve into the variations of CO_2_ adsorption in the presence of other gases, their selectivity of adsorption towards other gases, how the material's structure affects different gas molecules, and the changes in diffusion behaviours and interaction energies.

Studies have suggested that bare amorphous mesoporous silicas, like SBA-15 and MCM-41, exhibit larger CO_2_ adsorption capacities when compared with N_2_ and CH_4_, with the latter adsorbing to a larger extent as a result of stronger interactions with solids.^129,137^ Generally, CO_2_ is preferentially adsorbed over N_2_ at all temperatures, given the stronger dispersion interactions and larger quadrupole moment.^137^ In binary gas adsorption studies of CO_2_ and H_2_O, the material's lipophilicity affects the amount of water that gets adsorbed on the pore surface. Hydrophilic materials tend to adsorb more water, reducing adsorption and increasing diffusion of other gases. For hydrophobic surfaces, water molecules clump in the middle of the pore, which reduces CO_2_ diffusion and increases its adsorption.^148^ O_2_ in CO_2_/N_2_/O_2_ ternary mixtures has no effect on the selectivity of CO_2_ towards N_2_, and this is also observed in quaternary mixtures with additional H_2_O.^128^ Nonetheless, the presence of other gas molecules, even in small partial pressures, can significantly reduce CO_2_ adsorption if these molecules have preferential adsorption in the material's micropores.^148^ A possible workflow to perform computational studies on silica materials is presented in Fig. 12, encompassing the entire process from modelling to simulating gas mixtures.

In the case of PMO adsorbents, both experimental findings and computational results (using a DFT approach, see Table 3) unveiled their tendency to selectively adsorb CO_2_ rather than CH_4_. This feature positions them as prospective materials for separating these two gases.^48,122–125^ The incorporation of amino groups further enhanced the CO_2_/CH_4_ selectivity, with a more pronounced effect observed when using alkylamines.^48^ An M06-2X/cluster approach was employed to study the gas–host interactions between the additional components of the flue gas mixture and the PhPMO.^123^ Determining the adsorption energy of these gases plays a pivotal role in anticipating the uptake of CO, H_2_, N_2_, O_2_, NO, H_2_O, H_2_S, SO_2_, SO_3_, and NH_3_ by this material, eliminating the need for extensive Ph-PMO synthesis and numerous adsorption experiments. The preferential adsorption location for all these gases, also including CO_2_ and CH_4_, is near the silanols for this material. The energetic data reveal the preference of PhPMO for adsorbing CO_2_ over CO, CH_4_, N_2_, and H_2_, while NH_3_, H_2_O, SO_2_, SO_3_, and H_2_S exhibit higher adsorption energies than CO_2_, indicating their preferential adsorption over CO_2_.

## 6. Conclusion and perspectives

Technical and experimental advancements in the field of ssNMR and computational methods have enabled gaining insights into the molecular-level structure of porous surfaces, silica-based adsorbents. The progress made in studying host–gas interactions in the field of CO_2_ adsorption and separation has been greatly influenced by surface enhanced innovative techniques applied to sample preparation in controlled environments and the use of isotopically enriched gases and/or materials. The sample preparation can sometimes be difficult and hamper the obtained results (rigorous control of temperature, pressure and moisture levels is crucial) and good knowledge about the material textural properties (amount and type of functionalities, sample crystallinity and porosity, *etc.*) is important to correctly determine CO_2_ speciation. By combining computational tools, adsorption and ssNMR data, new chemisorbed and physisorbed CO_2_ species have been identified by controlling the former variables and working under controlled and reproducible conditions. Progressing beyond the state of the art requires getting closer to realistic gas adsorption conditions. In real world applications, the adsorption process is dynamic encompassing multiple gases flowing through the adsorbent material. *In situ* or operando NMR methods can be adapted to the study of adsorbents under real-time gas adsorption. Developing such methods is difficult for ssNMR applications as it requires a gas flowing through a spinning sample holder involving costly custom-made probe modifications. To circumvent this inconvenience, the use of 3D printing has become a popular and cost-effective way to fabricate components for NMR applications.^149–152^ Creating printed ssNMR components in-house will boost custom probe modifications, leading to new concepts and designs that can be tailored to specific applications. In the field of CO_2_ capture, we anticipate that 3D printing will play a crucial role, for instance in the adaptation of standard NMR probes for *in situ* flow MAS spectroscopy, in developing components for high-pressure rotor apparatus, and creating new gas-flow stator concepts (*e.g.*, breakthrough-style systems for studying gas separation).

As for computational methods, the integration of chemical information with artificial intelligence, through machine learning algorithms using, for instance, neural network prediction approaches will quickly become ubiquitous in structural determination in disordered systems as they offer considerable speed-up compared to traditional force fields, enabling simulations over longer time scales, typically ranging from nanoseconds to microseconds or more. MLPs are particularly useful for studying complex processes and large systems, as they can capture the underlying physics with reduced computational costs.^143,153^

As an example, Choudhary *et al.*^154^ have recently performed graph neural network predictions of the CO_2_ adsorption properties of hypothetical MOF materials, developing high accuracy and fast models for pre-screening applications. These tools offer new opportunities for predicting the structure of surface species in confined spaces and for optimizing distinct material properties.

## Conflicts of interest

There are no conflicts to declare.

## Supplementary Material
